# Antioxidant, antibacterial, *in vitro,* and *in silico* α-glucosidase inhibition activities and chemical profiling of *Usnea cornuta* Korb

**DOI:** 10.1371/journal.pone.0351423

**Published:** 2026-06-12

**Authors:** Deepa Karki, Anuraj Phunyal, Tika Ram Lamichhane, Deepika Karki, Achyut Adhikari

**Affiliations:** 1 Central Department of Chemistry, Tribhuvan University, Kirtipur, Kathmandu, Nepal; 2 Central Department of Physics, Tribhuvan University, Kirtipur, Kathmandu, Nepal; Universidade Federal do Para, BRAZIL

## Abstract

The total phenolic content and flavonoid content of the *Usnea cornuta* extract were evaluated as 210.31 ± 2.87 mg GAE/g and 22.42 ± 0.78 mg QE/g, respectively. The crude extract exhibited strong antioxidant activity (IC_50_: 32.91 ± 1.27 µg/mL) and notable anti-diabetic effects via α-glucosidase inhibition, with IC_50_ values of 2.59 ± 2.23 µg/mL for the dichloromethane extract. LC-MS analysis identified eleven metabolites like D-mannitol (1), galbinic acid (2), conhypoprotocetraric acid (3), roccellaric acid (4), diffractatic acid (5), haemathamnolic acid isomer (6), conprotocetraric acid (7), constictic acid I (8), salazinic acid II (9), menegazziaic acid (10), and one unknown compound (11). Among these, menegazziaic acid exhibited the strongest binding affinity of −9.7 kcal/mol with the target (PDB ID 3A4A), favorable molecular dynamics, binding free energy (MM/GBSA, and pharmacokinetic profiles. Furthermore, the extract showed strong antimicrobial activity, with inhibition zones of 23 mm and 26 mm at 10 mg/mL against *Staphylococcus aureus* ATCC 29213 and ATCC 245, respectively. These findings highlight the therapeutic potential of *Usnea cornuta*, specifically for managing oxidative stress, microbial infections, and type 2 diabetes.

## 1 Introduction

Diabetes mellitus, a global public health crisis, encompasses metabolic disorders characterized by persistent hyperglycemia due to insufficient insulin production, resistance, or both [1]. Affecting 537 million adults (10.5% of the 20–79 age group) in 2021, cases may rise to 643 million by 2030 and 783 million by 2045 [2]. The disease manifests as Type 1, involving autoimmune β-cell destruction, and Type 2, comprising over 90% of cases, marked by insulin resistance and inadequate secretion [3,4]. Emerging research suggests natural inhibitors, particularly from botanical sources, as potential modulators of α-amylase and α-glucosidase, offering promising, low-side-effect therapies [5].

Lichens, which are intricate life forms, showed a mutual relationship of fungi and algae, used for both nutritional and remedial purposes since antiquity [6]. They produce a wide range of secondary metabolites, particularly phenolic compounds that exhibit notable biological activity, including antioxidant properties [1,7–10]. Pharmacological interest in lichens is predominantly focused on their bioactive secondary metabolites, especially phenolic compounds, which are characterized by a reactive –OH group [11,12]. Furthermore, secondary metabolites derived from lichens have demonstrated diverse biological activities, including anti-oxidant, anti-inflammatory, analgesic, plant growth inhibition, enzyme inhibition, anti-proliferative, anti-cancer, anti-microbial, and cytotoxic effects [13–18].

The original specimen of *U. cornuta* (sensu stricto), characterized by a cornuta-type central mechanical axis (CMA) was selected for this study based on its ethnobotanical significance and therapeutic potential [19,20]. The presence of usnic acid in *Usnea* species, a bioactive compound traditionally used to manage metabolic disorders, including diabetes, suggests the potential antidiabetic relevance of this lichen [21]. Previous phytochemical investigations of *U. cornuta* have identified flavonoids, phenolics, and terpenoids, compounds well known for their antioxidant properties [22]. However, despite its traditional applications, scientific evidence supporting the antidiabetic and antioxidant activities of *U. cornuta* remains limited. Therefore, the present study aims to evaluate the antidiabetic potential of *U. cornuta* through integrated experimental and computational approaches.

Therefore, previous studies on *U. cornuta* have largely focused on taxonomic classification and preliminary phytochemical characterization with limited experimental and computational evidence linking its metabolites to diabetes-related biological activities [23,24]. This study presents the first systematic evaluation of the antibacterial, and α-glucosidase inhibitory activities, along with the identification of its bioactive compounds using LC-MS. It further reports the first application of molecular docking and molecular dynamics simulations to assess the interaction and stability of these compounds with α-glucosidase (PDB ID: 3A4A). The findings highlight *U. cornuta* as a promising natural source of digestive enzyme inhibitors relevant to hyperglycemia and diabetes management.

## 2 Experimental materials and methods

### 2.1 Chemicals required

High-quality organic solvents, Folin-Ciocalteu reagent (FCR), gallic acid, DPPH, quercetin, acarbose, DMSO, phosphate salts, Na_2_CO_3_, AlCl_3_, and other chemicals were obtained from Fisher Scientific. The α-glucosidase enzyme (*S. cerevisiae*) and substrate p-NPG were sourced from Sigma-Aldrich.

### 2.2 Assemble of lichen materials

Drawing upon literature references and expert knowledge, lichens identified as *U. cornuta* were collected from Champadevi (S1 Fig in S1 File) in Kathmandu, Nepal in January 2023, emphasizing their ethnobotanical significance. The specimens were examined by Research Officer Amrit KC at the National Herbarium and Plant Laboratories (KATH), Godawari, Lalitpur, Nepal, where a voucher specimen was deposited under voucher number *D. Karki, 20UC.* Following collection, lichen materials were meticulously shaded during drying, finely powdered, and subsequently stored in tightly sealed containers.

### 2.3 Preparation and fractionation of crude extracts

65 g of shade-dried *U. cornuta* powder was soaked in 90% methanol for three days with periodic agitation. The mixture was filtered, and the filtrates were concentrated using a rotary evaporation at 50 °C, followed by further drying in a water bath to obtain the crude extract. The extraction yield was calculated as a percentage of the initial material. The extract was fractionated into hexane, DCM, ethyl acetate, and water fractions. All solvent fractions were transferred to sterile glass vials, tightly sealed, and stored at −4 °C until further phytochemical and biological analyses.


$$\begin{array}{c} Percentage\;yield=\;\frac{Weight\;of\;extract\;(g)}{Weight\;of\;powder\;sample\;(g)}\times \text{1}\text{0}\text{0} \end{array}$$

### 2.4 Total phenolic content

The TPC in *U. cornuta*, was quantified using the FC reagent colorimetric method with slight modification [25]. In summary, 20 µL of the lichen sample, 100 µL of Folin-Ciocalteu reagent and 80 µL of Na_2_CO_3_ were mixed in a 96-well plate and incubated in the dark for 15 minutes, measured at 765 nm using a microplate reader (Epoch2, BioTek Instruments, Inc., USA).

### 2.5 Total flavonoid content

For the assessment of TFC, the aluminum trichloride method was utilized [26]. This entailed pipetting 20 µL of lichen extracts, 110 µL of distilled water, 60 µL of ethanol, 5 µL of potassium acetate, and 5 µL of AlCl_3_ into 96-well plates. The plates were then incubated for 30 minutes under light exclusion, and the absorbance was recorded at 415 nm.

### 2.6 DPPH scavenging activity

Various volumes of lichen fraction samples, increased in 100 µL increments, were added to individual wells of a 96-well plate, along with 100 µL of DPPH solution. The DPPH solution (0.1 mM) was prepared by dissolving 0.975 mg of DPPH in 25 mL of methanol and a stock solution of quercetin (100 µg/mL) for standard was prepared by dissolving 1 mg of quercetin in 10 mL of methanol. The plate was then incubated in the dark for 30 minutes, and the absorbance was measured at 517 nm. All measurements were performed in triplicate (n = 3) and analyzed using the following formula [27]. Additionally, the inhibition curve was generated using linear regression analysis in GraphPad Prism 8.0.


$$\begin{array}{c} \%\;Inhibition=\left(\frac{A_{control}-A_{sample}}{A_{control}}\right)\times \text{1}\text{0}\text{0} \end{array}$$

A_control =_ absorbances of the control

A_sample =_ absorbances of the sample solution

The software GraphPad Prism was employed to calculate the IC_50_ value.

### 2.7 *In-vitro* alpha-glucosidase inhibition activity

The inhibitory effect of *U. cornuta* on α-glucosidase was determined using p-NPG as the substrate, with slight modifications to the previous procedure [28]. In a 96-well plate, 20 µL of lichen extract, 10 µL of α-glucosidase (1 mg/mL; 0.625 Units/mL), and 130 µL of buffer were incubated at 37°C for 15 minutes, and the initial absorbance was measured at 405 nm. After the addition of 20 µL of 5 mM pNPG (3 mg in 2 mL buffer), incubation continued for 15 minutes, followed by the addition of 20 µL of Na_2_CO_3_ to develop a yellow color. The final absorbance was measured at 405 nm. Acarbose (1 mg/mL) was used as the standard inhibitor at concentrations ranging from 10 to 100 µg/mL to construct the inhibition curve, with all measurements performed in triplicate. The inhibition percentage was calculated using a standard formula and IC₅₀ values were determined using nonlinear regression analysis in GraphPad Prism 8.0.


$$\begin{array}{c} \%\;Inhibition=\left(\frac{A_{control}-A_{sample}}{A_{control}}\right)\times \text{1}\text{0}\text{0} \end{array}$$

A_control_ = absorbance of the control

A_sample_ = absorbance of the sample

GraphPad Prism was used to calculate IC_50_ value.

### 2.8 Anti-bacterial assay

This assay was tested against *Staphylococcus aureus* (ATCC 29213 and ATCC 245) and *Escherichia coli* (ATCC 25922) using the well-diffusion method. Bacterial cultures were grown in nutrient broth at 37 °C for 24 hours to reach 0.5 McFarland turbidity and were then spread onto Mueller-Hinton agar plates. Wells (6 mm) were filled with *U. cornuta* extract and incubated at 37°C for 18–24 hours. Zones of inhibition (ZOI) were measured in millimeters after observing clear inhibition zones. Cefoxitin and azithromycin were used as positive controls, while DMSO served negative control. Sensitivity was determined by inhibition zone diameters, with ≥23 mm (cefoxitin) and ≥25 mm (azithromycin) considered sensitive, 15–20 mm intermediate, and ≤15 mm resistant.

### 2.9 Instrumental LC-MS conditions

The ethyl acetate fraction of *Usnea cornuta* was subjected to liquid chromatography–mass spectrometry (LC-MS) analysis using a Shimadzu LC-MS 2020 system (Shimadzu Corporation, Japan). Chromatographic separation was carried out using a reversed-phase column housed in a CTO-20A column oven, maintained at 40°C, and samples were introduced via SIL-20A autosampler. The mobile phase consisted of 0.2% (v/v) formic acid in water (solvent A) and methanol (solvent B). A linear gradient elution program was employed over a total run time of 35 minutes flow rate of 0.4 mL/min(0–10 min, 90% A to 60% A; 10–25 min, 60% A to 10% A;25–30 min, held at 10% A; and 30–35 min, returned to initial conditions (90%A) for column re-equilibration). The injection volume for each sample was 1 µL. Mass spectrometric detection was performed using an electrospray ionization (ESI) source operated in both positive and negative ionization modes to ensure comprehensive metabolite profiling. The mass analyzer was set to scan a mass-to-charge (m/z) range of 50–700. The nebulizing gas flow rate was maintained at 1.5 L/min, and data acquisition was conducted under optimized default source parameters provided by the manufacturer. The chemical constituents present in the ethyl acetate fraction were tentatively identified based on a combination of retention times, mass spectral data, and high-resolution m/z values, followed by spectral interpretation and validation using MestReNova software. The obtained mass spectral features were compared with reported literature data to support compound annotation.

### 2.10 Computational experiment

#### 2.10.1 Preparation of ligands.

Based on LC-MS analysis, a total of 11 compounds (with one unidentified compound) were detected from *Usnea cornuta* extract. Among these, only 6 compounds were selected for docking due to the availability of their chemical structures in PubChem database portal [29]. The three-dimensional (3D) conformers of the selected ligands, along with acarbose (used as a standard compound for antidiabetic activity), were downloaded in SDF format. Energy minimization was performed for all ligands using the Avogadro v1.2 with the UFF force field, and the minimized structures were subsequently saved in PDB format for docking [30,31].

#### 2.10.2 Preparation of protein.

The crystal structure of alpha-glucosidase enzyme (PDB ID: 3A4A; resolution: 1.60 Å) was obtained from Protein Data Bank (www.rcsb.org) in PDB format. To ensure the structural integrity of the protein, validation was performed using SAVES v6.0 (https://saves.mbi.ucla.edu/) server [32]. Before molecular docking, all water molecules and co-crystallized ligands were removed. Kollman charges and polar hydrogens were incorporated using the AutoDock Tools v1.5.7 program [33]. The processed protein structure was saved in PDBQT format for docking analysis.

#### 2.10.3 Molecular docking.

Molecular docking was performed to investigate the interactions between selected inhibitors with alpha-glucosidase. The AutoDock Vina v1.2 was employed for molecular docking, and resulting complexes were visualized through the PyMOL v2.4.0 software [34,35]. The grid box was 35 Å × 35 Å × 35 Å, with its center at the coordinates X = 23.244385, Y = −7.482308, and Z = 23.570923. All docking parameters were kept at their default values, except for exhaustiveness, which was increased to 48 to improve search accurate, and the number of modes was set to 20. The detailed procedure outlined by Karki et al. [36].

#### 2.10.4 Validation of docking protocol.

The protocol was validated by re-docking the co-crystalized ligand into protein’s active site using PyMOL. Literally, a low root mean square deviation (RMSD) (below 2 Å) between re-docked and native ligand value indicates the reliability of docking protocol [37]. Furthermore, the protein’s active site was identified using the CASTp server [38].

#### 2.10.5 Molecular dynamics procedure.

Molecular dynamics simulations of the protein-ligand complexes were performed using GROMACS 2019.6 program [39]. The CHARMM36 force field was applied to receptor topology [40]. Ligand topologies were generated via SwissParam server [41]. The system was solvated in a cubic TIP3P water box. NaCl (0.15 M) ions were added to neutralize the system. Energy minimization was conducted using the steepest descent algorithm. Subsequently, the system was equilibrated in two phases: 100 ps NVT ensemble (310 K) using a modified Berendsen thermostat, followed by 100 ps NPT ensemble using the Berendsen method. Long-range electrostatic interactions were treated using the Particle Mesh Ewald (PME) method with 30 Å cutoff [42]. After equilibration, a 100 ns production run was conducted with 1000 frames at 2 fs time step. Geometrical parameters, including RMSD, RMSF, Rg, SASA, hydrogen bonding, and FELs were analyzed using GROMACS modules, while PCA and DCCM were assessed through the R programming package Bio3D. A detailed protocol was mentioned by Gyawali et al [43].

#### 2.10.6 Binding free energy calculations.

The binding free energies for protein-ligand complexes were calculated by using the MM/GBSA method facilitated by the gmx-MMPBSA program v1.5.7 [44,45]. For each system, 200 frames extracted from the final, stable 20 ns of 100 ns production trajectory for energy calculations. The methodology closely followed the protocols described by Genheden et al. [46].

#### 2.10.7 Drug-likeness profiles.

ADMET analysis of menegazziaic acid was performed using the ProTox-3.0 and SwissADME server [47,48].

## 3 Results

### 3.1 Percentage yield

The percentage yield of *U. cornuta* in 90% methanol was 16.21% and the yield of fractions like hexane, DCM, ethyl acetate and water extracted from the crude extract of ethanol were 40.32%, 27.41%, 29.79% and 9.01% respectively (Table 1).

**Table 1:** Percentage yield of U. cornuta lichen extract.

The scientific name of Lichen	Weight of lichen sample (g)/Starting sample	Weight of extract (g)/Fraction	Percentage yield (%)
*U. cornuta*	65	10.54	16.21
*Hexane*	10.54	4.25	10.32
*Dichloromethane*	10.54	2.89	27.41
*Ethyl acetate*	10.54	3.14	29.79
*Water*	10.54	0.95	9.01

### 3.2 Estimation of total phenolic content and total flavonoid content

The total phenolic content (TPC) in *U. cornuta* extracts was quantified as 210.31 ± 2.87 mg GAE/g using gallic acid calibration (Y = 0.0054x + 0.0188, R^2^ = 0.9901). A 96-well plate method facilitated this analysis. The total flavonoid content (TFC) was determined as 222.42 ± 0.782 mg QE/g using quercetin calibration (Y = 0.0183x + 0.0037, R^2^ = 0.9926) (Table 2).

**Table 2:** Total phenolic content and total flavonoid content of the crude extract of U. cornuta.

Estimation	*U. cornuta*
Total phenolic content (mg GAE/g)	210.30 ± 2.87
Total flavonoid content (mg QE/g)	222.42 ± 0.782

### 3.3 Calculation of anti-oxidant and α-glucosidase inhibition activity

The free radical scavenging activity of *U. cornuta* lichen extract showed an IC_50_ of 32.91 ± 1.26 µg/mL, comparable to quercetin (31.44 ± 0.21 µg/mL) with standard curve (S2 Fig in S1 File). Among fractions, ethyl acetate fraction exhibited the highest activity (IC_50_ = 12.75 ± 1.13 µg/mL), whereas water showed the weakest activity (IC_50_ = 77.05 ± 0.90 µg/mL) followed by hexane (IC_50_ = 44.04 ± 0.93 µg/mL) and DCM (IC_50_ = 13.81 ± 0.60 µg/mL).

Similarly, α-glucosidase inhibition revealed an IC_50_ of 13.15 ± 1.24 µg/mL for the crude extract. Fraction IC_50_ values ranged from hexane (0.37 ± 0.17 µg/mL), DCM (2.59 ± 2.23 µg/mL) and ethyl acetate (3.72 ± 0.64 µg/mL), with acarbose used as the standard with curve (S3 Fig in S1 File) (3.11 ± 0.22 µg/mL), presented in Table 3 and the activities were performed in triplicate form (n = 3) (Table 3). These values are tally in 95% confidence interval which showed p values are within the range (0.001–0.0175), reflects p value are below 0.05.

**Table 3:** IC_50_ value of fraction of U. cornuta for anti-oxidant and α-Glucosidase inhibition activity.

Fractions	Anti-oxidant IC_50_ (µg/mL)	P values	α*-Glucosidase* IC_50_ (µg/mL)	P values
Crude	32.91 ± 1.26	0.0001	13.15 ± 1.24	<0.0001
Hexane	44.04 ± 0.93	<0.0001	0.37 ± 0.17	0.0018
DCM	13.81 ± 0.60	0.0175	2.59 ± 2.23	0.0014
Ethyl acetate	12.75 ± 1.13	<0.0001	3.72 ± 0.64	0.0113
Water	77.05 ± 0.90	0.0005		–
**Quercetin**	31.44 ± 0.21	<0.0001	_	–
**Acarbose**	–	–	3.11 ± 0.22	<0.0001

The mean ± standard error mean (SEM) is used to represent data.

### 3.4 Antibacterial assay

**Fig 1:**
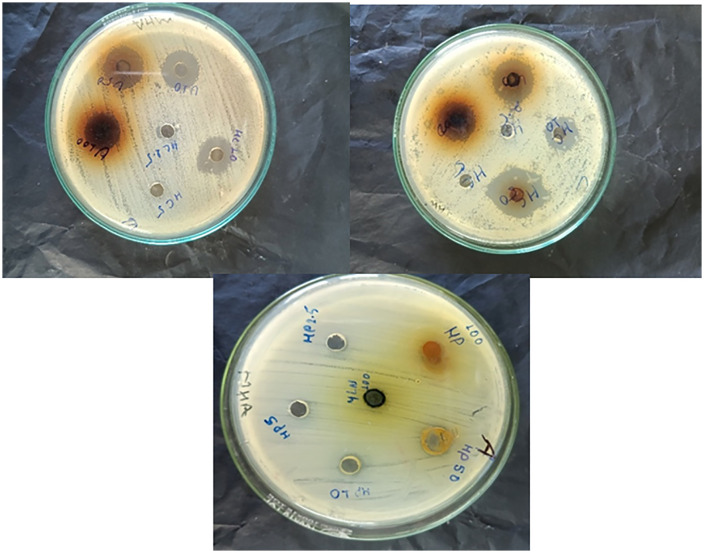
Zone of Inhibition against (a) *Staphylococcus aureus* (ATCC 29213), (b) *Staphylococcus aureus* (ATCC 245), and (c) Escherichia coli (ATCC 25922) at 10 mg/mL (L1) and 5 mg/mL (L2).

Following treatment and incubation of the inoculated plates for 24 hours, antibacterial activity of the methanolic extract of *U. cornuta* was observed against *Staphylococcus aureus* ATCC 29213 and *Staphylococcus aureus* ATCC 245. However, no activity was observed against *Escherichia coli* ATCC 25922. The antibacterial screening results are shown (Fig 1).

Both concentrations of the methanolic extract, 10 mg/mL (L1) and 5 mg/mL (L2), exhibited activity against the microorganism strains. The crude extract inhibited the growth of *S. aureus* ATCC 29213 with a zone of inhibition (ZOI) of 23 mm at 10 mg/mL and 19 mm at 5 mg/mL, while *S. aureus* ATCC 245 showed ZOI of 26 mm and 16.5 mm at the same concentrations. However, no activity was recorded against *E. coli* ATCC 25922 (S1 Table in S1 File).

### 3.5 Identification of metabolites in lichen *Usnea cornuta* by LC-MS

**Fig 2:**
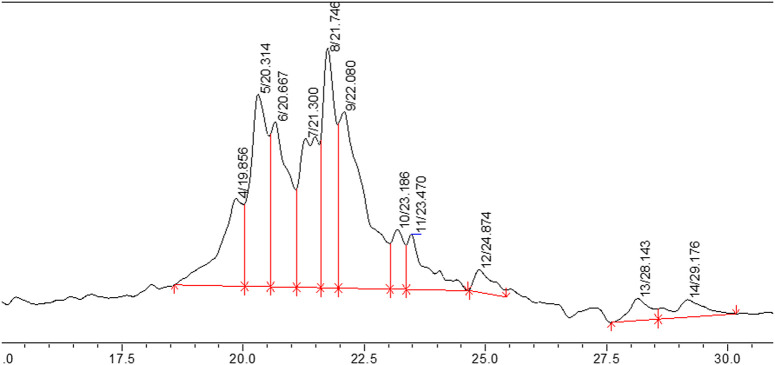
Total Ion Chromatogram (TIC) of *U. cornuta* in+ ve ESI mode.

**Fig 3:**
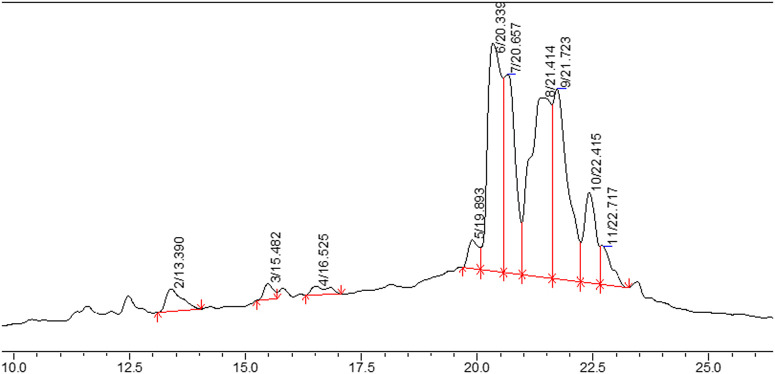
Total Ion Chromatogram (TIC) of *U. cornuta* in -ve ESI mode.

The LC-MS analysis of *U. cornuta* hexane extract identified metabolites in positive and negative ESI modes, generating total ion chromatograms (TICs) (Figs 2 and 3). Based on accurate masses, retention times, standards, and database searches (Mest ReNova), presence of 11 compounds was confirmed in the ethyl acetate extract (S2 Table in S1 File). In positive ion mode, a peak at m/z 181.0708 (C_6_H_13_O_6_) with a retention time of 32.885 minutes was identified as d-mannitol, a major component in *Coffea Arabica L.* pulp [49]. Another peak at m/z 429.0459 (C_20_H_13_O_11_) with a retention time of 20.314 minutes was identified as galbinic acid, compound found in *E. trulla* (S3 Table in S1 File) [50,51].

Additionally, in this study, conhypoprotocetraric acid (m/z 359.0762, C_18_H_15_O_8_, Rt. 13.250 min) was identified as a depsidone, and has been previously reported in *Ramalina siliquosa* [52]. Roccellaric acid (m/z 325.2380, C_19_H_33_O_4_, 32.995 min) was detected in negative ion mode and classified as a cycloaliphatic acid [53], while diffractaic acid (m/z 373.1292, C_20_H_21_O_7_, 20.339 min) was detected as depsides [54]. Another compound, menegazziaic acid (C_18_H_13_O_9_), was also detected with *m/z* 373.0555 and retention time of 20.657 minutes. This discovery aligns with information reported in a previous research document regarding *Usnea longissima* and the genus *Usnea* [50,55]. Additionally, a peak with a mass-to-charge ratio (*m/z*) of 403.0660, retention time of 15.482 minutes, and molecular formula of C_19_H_15_O_10_ was identified as haemathamnolic acid isomer, categorized as a depside compound.

This compound has been reported as a bioactive compound of *Pseudevernia furfuracea* [56]. In addition, a depsidones compound, salazinic acid II (C_18_H_11_O_10_), was detected with an *m/z* of 387.0348 and a retention time of 16.525 minutes in negative mode [19]. Conprotocetraric acid, classified as a depsidones compound with a molecular formula of C_18_H_13_O_9_, was eluted at 19.893 minutes and detected at *m/z* 375.0719. This compound has been found similarly in *Parmotrema* species [57]. Additionally, constictic acid I, with a molecular formula of C_19_H_13_O_10_, appeared with a retention time of 22.415 minutes at an *m/z* peak of 401.0513 in negative ion mode. These identifications were made through comparison with previously recognized reported compounds [19]. Furthermore, an unidentified compound with the molecular formula C_20_H_13_O_12_ was also detected as a peak at *m/z* 445.0410, with a retention time of 23.186 minutes in positive ion mode [19].

### 3.6 Validation of protein structure and docking protocol

**Fig 4:**
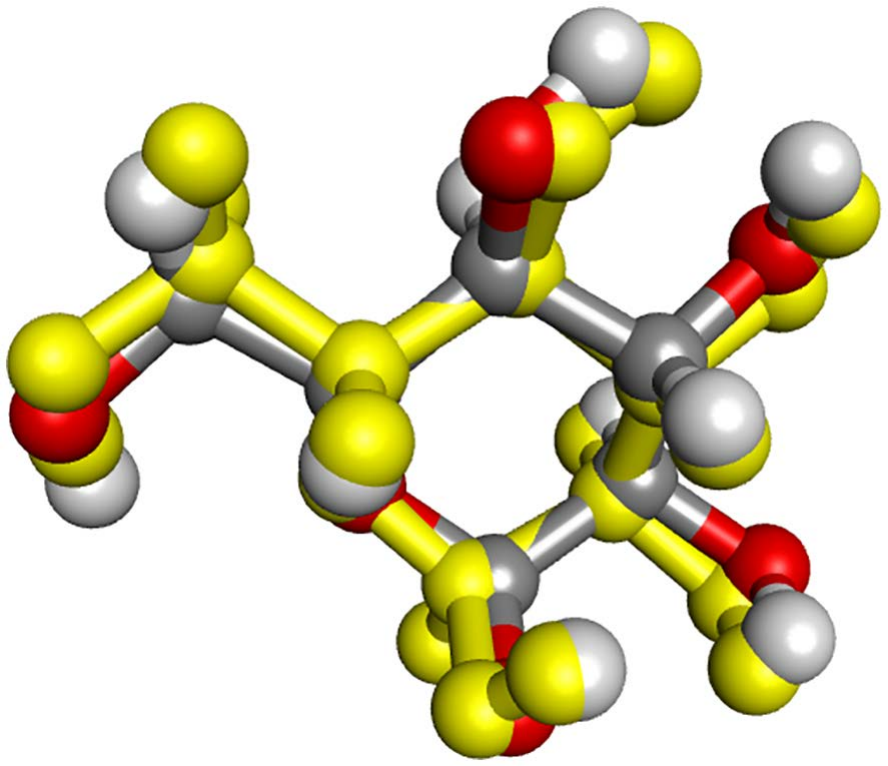
Docking validation. Superimposition of native ligand (from PDB, grey) and the re-docked one (yellow) ensures an RMSD of less than 2 Å (ball and stick model).

The overall quality factor of 93.7716 indicates good quality of protein model, and this was further supported by the Ramachandran plot generated through the PROCHECK module, which showed acceptable stereo-chemical properties (S4 Fig in S1 File). On other hand, RMSD between docked pose and original crystallographic position was found to be 1.943 Å, hinting that the parameters were valid for other ligands [58] (Fig 4).

### 3.7 Molecular docking analysis

**Fig 5:**
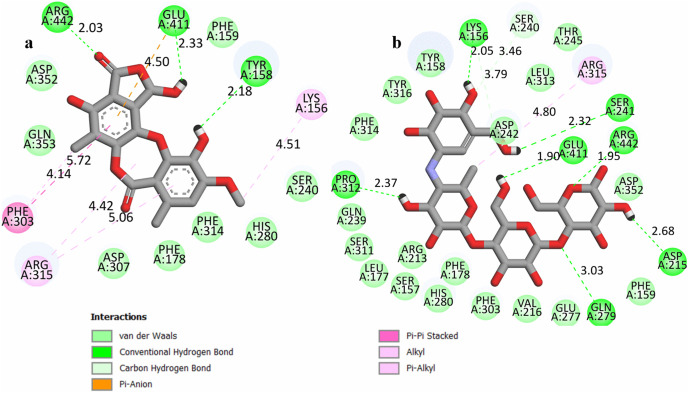
2D binding interaction of protein with ligands. (a) menegazziaic acid and (b) acarbose with protein (PDB ID 3A4A).

Molecular docking results identified menegazziaic acid as a potent alpha-glucosidase inhibitor, with a binding affinity of −9.7 kcal/mol against protein (PDB ID: 3A4A), surpassing acarbose, which has an affinity of −8.9 kcal/mol (Table 4). These negative binding affinity values indicate the formation of stable protein-ligand complexes within the enzyme’s active site. Interaction analysis performed using the Biovia Discovery 2021, which revealed that menegazziaic acid formed multiple interactions in orthosteric and allosteric sites, including hydrogen bonds, π-π stacking, and van der Waals’ interactions (involvement of active site residues) with key amino acid residues such as TYR158 and GLU411, ARG442 (Table 5) [59]. The results supported reported article [60,61]. Similarly, acarbose showed diverse interactions with the enzyme, involving residues such as GLU411, ARG442, and LYS156 (Fig 5). The binding affinities and docking poses suggested that both compounds effectively bind to the active and allosteric sites of protein; however, menegazziaic forms a more stable interactions, particularly at allosteric site, highlighting its potential as a promising anti-diabetic candidate, warranting further stability testing and clinical validation.

**Table 4:** Docking score of ligands.

S.N	PubChem CID	Compound Name	Binding affinity (k/cal/mol)
**1.**	71438918	Menegazziaic acid	**−9.7**
**2.**	101286191	Galbinic acid	−8.7
**3.**	274259652	Conprotocetraric acid	−8.6
**4.**	94870	Diffractaic acid	−8.1
**5.**	10019295	Roccellaric acid	−6.3
**6.**	6251	D-mannitol	−5.8
**7.**	41774	Acarbose	**−8.9**

**Table 5:** Different types of interactions of menegazziaic acid and acarbose with protein (PDB ID 3A4A).

Compounds	Types of interactions	Interacted residues with distance (Å)
Menegazziaic acid	H-bonding	TYR158 (2.18), GLU411 (2.33), (4.50), ARG442 (2.03)
	Alkyl, Pi-Alkyl, Pi-Anion, Pi-Pi Stacked	LYS156 (4.51), ARG315 (4.42), (5.06), PHE303 (4.14), (5.72), GLU411 (4.50)
	van der Waals’	PHE159, PHE178, SER240, HIS280, ASP307, PHE314, ASP352, GLN353
Acarbose	H-bonding	LYS156, ASP215, SER241, GLN279, PRO312, GLU411, ARG442
	Alkyl, Pi-Alkyl, Pi-Anion, Pi-Pi Stacked	ARG315 (4.80)
	van der Waals’	PHE303, VAL216, GLU277, PHE159, ASP352, ASP242, LEU313, THR245, SER240, TYR158, TYR316, PHE314, GLN239, SER311, LEU177, ARG213, PHE178, HIS280

### 3.8 Molecular dynamics simulation (MDS)

#### 3.8.1 Root mean square deviation (RMSD).

**Fig 6:**
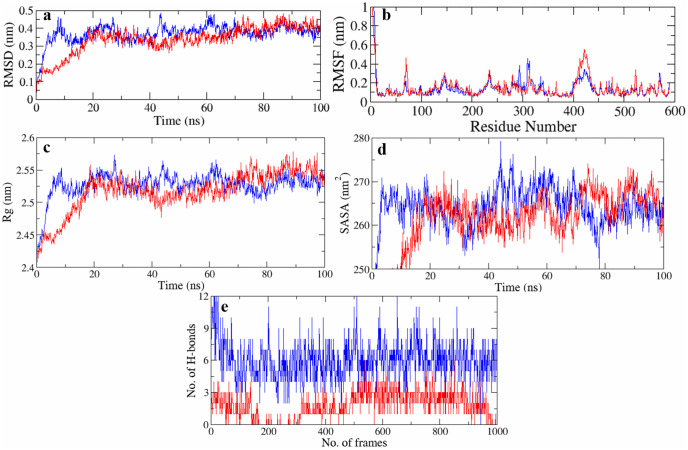
Different plots derived from 100 ns MDS trajectories of the complexes. **(a)** RMSD plot of menegazziaic acid and acarbose relative to the protein backbone, **(b)** RMSF curves of alpha carbon atoms, **(c)** Rg curves of protein, **(d)** SASA plot of the protein, and **(e)** the count of hydrogen bonds that bind ligands to the protein. Red curve = menegazziaic acid, blue curve = acarbose.

The RMSD curve, derived from molecular dynamics simulations trajectory, was used to assess conformational stability of the protein-ligand complexes (Fig 6a) [62]. The protein with menegazziaic acid adduct showed an average RMSD of 3.37 ± 0.77 Å, whereas the protein- acarbose complex exhibited a slightly higher value of 3.71 ± 0.51 Å. Both adducts reached equilibrium at around 20 ns. After equilibration, the RMSD values stabilized at approximately 3.5 Å, with minor fluctuations of about 0.5 Å. Notably, similar results have been reported in previous studies [63]. These results indicate that the ligands consistently maintained their docked positions throughout the molecular dynamics simulation period in both complexes.

#### 3.8.2 Root mean square fluctuation (RMSF).

RMSF analysis was performed to assess the flexibility of amino acid residues during MDS [64]. In both protein-menegazziaic acid and protein-acarbose adducts, the majority of amino acid residues exhibited RMSF values below 2 Å, reflecting limited structural fluctuations (Fig 6b). In contrast, residues located between PRO400 to ARG450 displayed higher peaks around 3 Å to 5 Å, hinting at increased mobility in this region. This elevated flexibility may be attributed to a deficiency of alpha or beta-pleated sheet structures in this region [37].

Comparative analysis revealed that the protein-menegazziaic acid exhibited an average RMSF of 1.40 ± 1.01 Å, while the protein-acarbose complex showed less fluctuation compared to protein-menegazziaic acid complex, with a slightly lower value of 1.36 ± 1.07 Å, suggesting reduced flexibility of the protein-acarbose complex and indicating its higher stability [65]. Similar trend have been reported in previous article [66].

#### 3.8.3 Radius of gyration (R_g_).

Rg profiles of both protein-menegazziaic acid and protein-acarbose complexes were monitored throughout the simulation period to assess structural compactness [67]. The average values were 2.49 ± 0.03 nm and 2.52 ± 0.01 nm, respectively. The protein-menegazziaic acid complex stabilized around 20 ns, whereas the protein-acarbose complex stabilized earlier, about 10 ns. Both complexes exhibited minor fluctuations of approximately 0.5 nm, indicating minimal structural expansion or contraction (Fig 6c). throughout the simulation period, consistent with previously reported studies (Fig 6c) [68,69]. Top of FormBottom of FormTop of FormBottom of Form

#### 3.8.4 Solvent accessible surface area (SASA).

The SASA of protein in manegazziaic acid adduct was around 261.25 ± 7.04 nm^2^, while the protein-acarbose complex showed a slightly higher value of 263.95 ± 4.98 nm^2^. The complex values ranged from 265 to 270 nm^2^ after 10 ns of simulation (Fig 6d). The relatively smooth SASA profiles inferred the condensed structure of protein-ligand adducts [70].

Furthermore, the SASA analysis suggests that both menegazziaic and acarbose remained stable within the protein’s orthosteric and allosteric sites without exposing the hydrophobic portion [71].

#### 3.8.5 Hydrogen bonds.

The number of hydrogen bonds was monitored over 1,000 frames during the molecular dynamics simulations (Fig 6e). The protein-menegazziaic acid adduct maintained about two hydrogen bonds beyond 500 frames, whereas the protein-acarbose complex sustained around six hydrogen bonds beyond 200 frames, indicating greater stability of the acarbose at the orthosteric and allosteric sites. Overall, the results suggest stable protein-ligand interactions with minimal RMSD fluctuations throughout the simulation period [72].

#### 3.8.6 PCA, DCCM, and FEL analysis.

**Fig 7:**
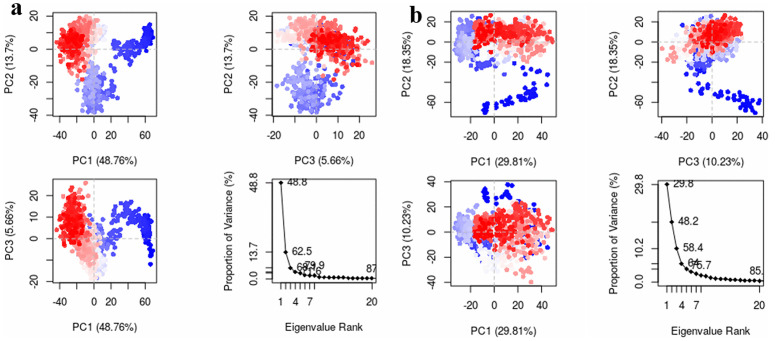
Eigenvalue distribution from PCA showing the proportion of variance. (a) menegazziaic acid, (b) acarbose complexes. Different colors (blue, white, and red indicate different stages (initial, intermediate, and final).

Principle component analysis (PCA) and dynamic cross-correlation matrix (DCCM) analyses were performed using Bio3D-R Package taking 100 ns MDS trajectories to evaluate the collective motion and correlated residue dynamics of the protein in complex with ligands. In PCA analysis, the protein-menegazziaic acid showed the highest contribution to structural variance in PC1 cluster (48.76%), followed by PC2 (13.7%), and PC3 (5.66%) clusters (Fig 7a). This high concentration of variance suggests that the protein moves in a specific, large-scale manner when bound to menegazziaic acid.

In contrast, the protein-acarbose complex showed significantly lower variance along PC1 (29.81%), PC2 (18.35%), and PC3 (10.23%) clusters, respectively (Fig 7b), indicating that acarbose binding makes the protein more flexible in many ways. In addition, the eigenvalue distribution confirmed that PC1 dominates in capturing the major conformational dynamics of both complexes.

**Fig 8:**
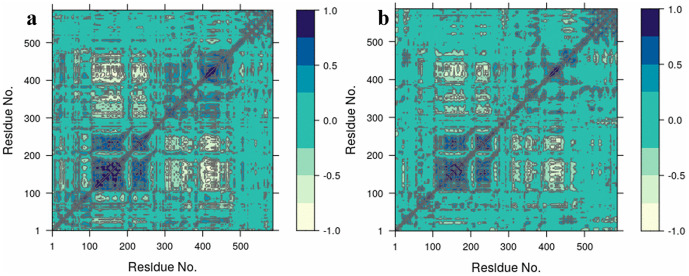
DCCM plots illustrating correlated residue motions, depicted in blue and sea green colors, respectively. (a) menegazziaic acid, (b) acarbose complexes.

Residue-residue correlation effects of the amino acid were analyzed using by DCCM plots. The protein-menegazziaic acid complex showed a maximum positive interaction distribution (0–1) across functional regions more clearly defined anti-correlated motions between distant domains. This indicates that menegazziaic acid stabilizes a tightly coupled dynamic state, which may facilitate allosteric transitions more smoothly (Fig 8a). In contrast, protein-acarbose complex displayed a mixture of strong positive and negative correlations (−1–0), suggesting more constrained and coordinated pattern of residue motion (Fig 8b).

**Fig 9:**
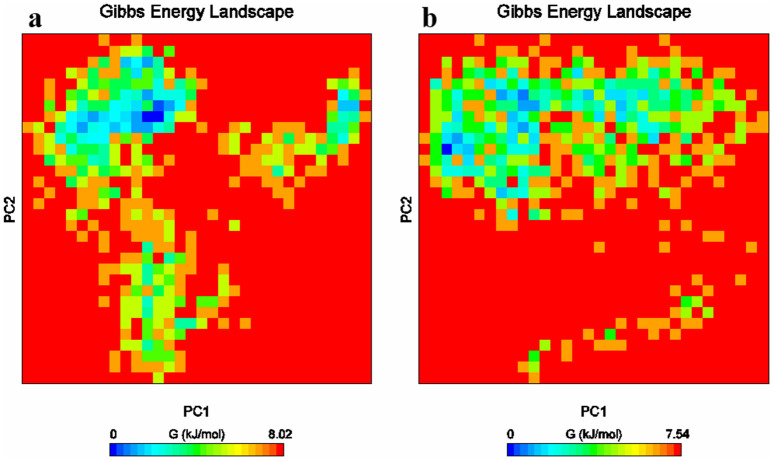
Free energy landscape based on PC1 and PC2. Different colors represent varying energy levels. (a) menegazziaic acid and (b) acarbose complexes.

The free energy landscapes (FELs) of the protein-ligand complexes were evaluated using the first two principal components (PC1 and PC2). The protein-menegazziaic acid complex has multiple low-energy basins, which implies that protein moves across conformational sub-states separated by energy barriers of approximately 8.02 kJ/mol (Fig 9a). In contrast, the protein-acarbose complex showed more compact and well-defined FEL with low-energy basins, indicating conformational ensemble or a transition towards prevailing state. The minimum free energy reached roughly 7.54 kJ/mol, suggesting that its energy surface is a little flatter than protein-menegazziaic acid complex (Fig 9b).

Overall, the PCA, DCCM, and FEL analyses demonstrate that menegazziaic acid promotes greater conformational flexibility and dynamic residue correlations, whereas acarbose induces more rigid and dynamically coordinated protein structure.

#### 3.8.7 Result from MM/GBSA calculations.

**Fig 10:**
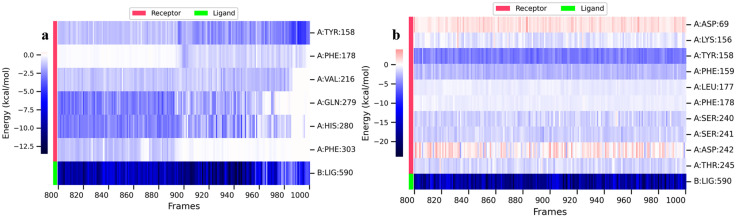
Heat map of complexes. (a) menegazziaic acid and (b) acarbose with amino acid during the final 200 frames.

The MM/GBSA binding free energy was determined as −15.79 ± 4.39 kcal/mol for the protein- menegazziaic acid complex and −28.64 ± 4.34 kcal/mol for the protein-acarbose complex (S3 Table in S1 File). The negative values indicate a spontaneous binding process, consistent with the docking results, and favorable product formation [73,74]. Heat maps (200 frames) highlighted the influence of ligands on both complexes and showed significant ligand influence on both complexes, with dark blue regions indicating key residues, while lighter blue represents other amino acids (Fig 10). Furthermore, last 200 frames of equilibrated trajectories and per-residue energy components are depicted (S5 and S6 Figs in S1 File). These results confirm the correlation between docking interactions and binding energy calculations.

#### 3.8.8 Drug-likeness Properties.

**Fig 11:**
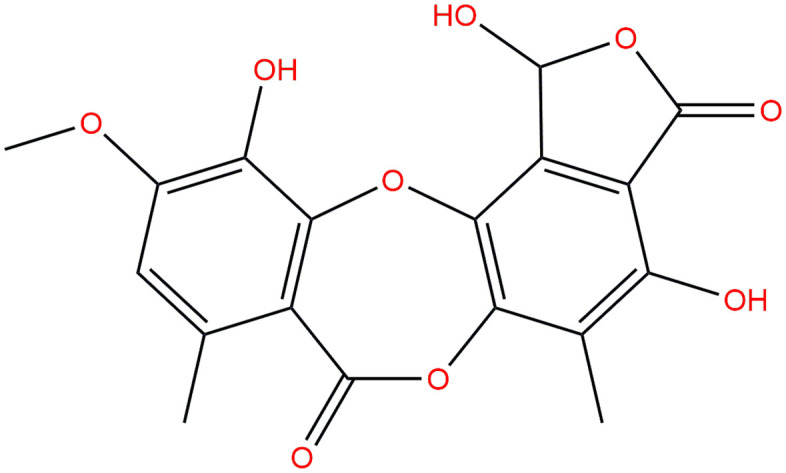
Structure of menegazziaic acid.

The menegazziaic acid lies in toxicity class IV compounds with predicted LD_50_ 962 kg/mg using ProTox 3.0 server. Various parameters are crucial for the selection of an optimal drug candidate, with physicochemical properties playing a significant role in reliable design of drugs [75] (S4 Table in S1 File). Its total polar surface area (131.75 Å), significantly lower than acarbose (321.17 Å), indicating better cell membrane permeability. Menegazziaic acid adheres to Lipinski’s rule of five and has a bioavailability score of 0.55, suggesting favorable oral absorption. It does not cross the blood-brain barrier (BBB) and exhibits low gastrointestinal absorption. These attributes, along with its physicochemical properties, position menegazziaic acid as a potential candidate for diabetes treatment (Fig 11).

## 4 Discussion

*Usnea* species are well known for producing a wide range of secondary metabolites with diverse biological activities [76]. In the present study, *Usnea cornuta* exhibited notably high total phenolic content (TPC) and moderate total flavonoid content (TFC), which may partly explain its strong antioxidant and enzyme-inhibitory activities. While previous reports on other *Usnea* species have shown wide variability in phenolic and flavonoid levels, these differences are likely attributable to species-specific metabolic profiles and environmental factors rather than direct indicators of biological potency [72,73].

Among the solvent fractions, ethyl acetate and hexane fractions demonstrated the strongest antioxidant and α-glucosidase inhibitory activities, respectively. This pattern is consistent with the preferential extraction of lipophilic lichen metabolites, such as usnic acid, salazinic acid, and related depsides and depsidones, which are known to exhibit antioxidant, enzyme-inhibitory properties, and antimicrobial [74–79]. The remarkably low IC₅₀ value of hexane fraction against α-glucosidase suggests that non-polar constituents play major role in enzyme inhibition, likely through favorable interactions with hydrophobic regions of the enzyme active site.

An antibacterial activity of *U. cornuta* also correlated well with its chemical profile. The pronounced inhibition zones observed against *Staphylococcus aureus* strain are in line with the presence of well-established lichen acids, such as usnic acid and salazinic acid, which have been widely reported to disrupt bacterial cell membranes and metabolic processes [80,81]. Important chemical constituents are present in *Usnea* species, LC-MS profiling identified multiple metabolites, including a tentatively assigned unknown compound, further supporting chemical diversity contributing to the observed antimicrobial effects, antioxidant and α-glucosidase inhibitory activities [82].

Molecular docking analysis further supported these findings, as selected lichen metabolites demonstrated strong binding affinities toward α-glucosidase, indicating potential competitive inhibition. Compounds were selected for docking based on confident LC-MS identification, documented occurrence in *Usnea* species, and structural features relevant to enzyme inhibition. Menegazziaic acid was highlighted in this study based on prior literature identifying it as an important secondary metabolite in several *Usnea* species and its favorable in silico interaction with α-glucosidase [83]]. Its presence in *U. cornuta* was tentatively suggested by LC–MS mass analysis; however, it was not quantitatively determined. Therefore, menegazziaic acid should be regarded as a computationally prioritized candidate rather than a confirmed major constituent.

Although direct interactions with catalytic dyad (only Van der Waals’) were not observed, the strong binding affinity and engagement of functionally important residues suggested a potential allosteric inhibition mechanism, which warrants further experimental validation.

## 5 Conclusions

This study evaluated methanol extracts of *U. cornuta* for antioxidant, antidiabetic, and antibacterial activities. Analyses of total phenolic and flavonoid content revealed significant inhibitory effects against DPPH radicals, α-glucosidase, and bacterial strains. Among the solvent fractions, ethyl acetate fraction showed the strongest antioxidant activity, while hexane fraction demonstrated the best α-glucosidase inhibition. LC-MS analysis identified several bioactive compounds, including menegazziaic acid, which *in silico* analysis suggests as a promising therapeutic option for diabetes. These findings highlight *U. cornuta*’s potential for managing oxidative stress, hyperglycemia, and bacterial infections. Future work will isolate and characterize active compounds, explore mechanisms of action, and conduct *in vivo* and clinical studies.

## Supporting information

S1 File S1 Fig. Geographical map of collection area, Champadevi, Kathmandu, Nepal. **S2 Fig.** Standard curve of DPPH inhibition by Quercetin. **S3 Fig.** α-Glucosidase inhibition activity of Acarbose. **S4 Fig.** Ramachandran plot of protein (PDB ID: 3A4A). **S5 Fig.** Changes in the binding free energy of different protein adducts with (a) protein-menegazziaic acid and (b) acarbose, red indicates the moving average. **S6 Fig.** Residues contribution in binding energies (kcal/mol) of complexs. (a) menegazziaic acid and (b) acarbose complexes. **S1 Table.** Zone of inhibition of crude extract of *U. cornuta* against bacteria strain. **S2 Table.** Identification of metabolites in lichen *Usnea cornuta* by LC-MS. **S3 Table.** Mass spectra chromatogram of 11 compounds present in *U. cornuta*. **S4 Table.** Change in binding energies (kcal/mol) of complex with different components. (a) menegazziaic acid and (b) acarbose complexes. **S5 Table.** Drug-Likeness Properties of menegazziaic acid and acarbose through the Swiss ADME Portal.
(ZIP)
