# Supplementary material for: Antioxidant, antibacterial, in vitro, and in silico α-glucosidase inhibition activities and chemical profiling of Usnea cornuta Korb
Source: PLoS One. 2026 Jun 12;21(6):e0351423. doi: 10.1371/journal.pone.0351423 (PMC13262885; doi:10.1371/journal.pone.0351423)
Supplement: S1 File — S2 Fig. Standard curve of DPPH inhibition by Quercetin. S3 Fig. α-Glucosidase inhibition activity of Acarbose. S4 Fig. Ramachandran plot of protein (PDB ID: 3A4A). S5 Fig. Changes in the binding free energy of different protein adducts with (a) protein-menegazziaic acid and (b) acarbose, red indicates the moving average. S6 Fig. Residues contribution in binding energies (kcal/mol) of complexs. (a) menegazziaic acid and (b) acarbose complexes. S1 Table. Zone of inhibition of crude extract of U. cornuta against bacteria strain. S2 Table. Identification of metabolites in lichen Usnea cornuta by LC-MS. S3 Table. Mass spectra chromatogram of 11 compounds present in U. cornuta. S4 Table. Change in binding energies (kcal/mol) of complex with different components. (a) menegazziaic acid and (b) acarbose complexes. S5 Table. Drug-Likeness Properties of menegazziaic acid and acarbose through the Swiss ADME Portal. (ZIP) [file pone.0351423.s001.zip › Supporting Information/S2 Table.docx]

**S2 Table. Identification of metabolites in lichen *Usnea cornuta* by LC-MS**

| **S.N.** | **Identified compounds** | **Classification** | **Observed (*m/z*) (g/mol)** | **Calculated mass (*m/z*) (g/mol)** | **Product ions (*m/z*)** | **Retention Time** |
| --- | --- | --- | --- | --- | --- | --- |
| **1** | D- mannitol  C_6_H_13_O_6_ | Carbohydrates | 181.0708 | 181.0712 | [M+H]^+^ | 32.885 |
| **2** | Galbinic acid  C_20_H_13_O_11_ | Depsidones | 429.0459 | 429.0458 | [M+H]^+^ | 20.314 |
| **3** | Conhypoprotocetraric acid  C_18_H_15_O_8_ | Depsidones | 359.0762 | 359.0767 | [M+H]^+^ | 13.25 |
| **4** | Roccellaric acid  C_19_H_33_O_4_ | Cycloaliphatic acids | 325.2380 | 325.2384 | [M+H]^-^ | 32.995 |
| **5** | Diffractatic acid  C_20_H_21_O_7_ | Depsides | 373.1292 | 373.1287 | [M+H]^-^ | 20.339 |
| **6** | Haemathamnolic acid isomer  C_19_H_15_O_10_ | Depsides | 403.0660 | 403.0665 | [M+H]^-^ | 15.482 |
| **7** | Conprotocetraric acid  C_18_H_13_O_9_ | Depsidones | 375.0719 | 375.0716 | [M+H]^-^ | 19.893 |
| **8.** | Constictic acid I  C_19_H_13_O_10_ | Depsidones | 401.0513 | 401.0509 | [M+H]^-^ | 22.415 |
| **9** | Salazinic acid II  C_18_H_11_O_10_ | Depsidones | 387.0348 | 387.0352 | [M+H]^-^ | 16.525 |
| **10** | Menegazziaic acid  C_18_H_13_O_9_ | Depsidones | 373.0555 | 373.0560 | [M+H]^-^ | 20.657 |
| **11** | Unknown  C_20_H_13_O_12_ | Unidentified | 445.0410 | 445.0407 | [M+H]^+^ | 23.186 |
