# Supplementary material for: Antioxidant, antibacterial, in vitro, and in silico α-glucosidase inhibition activities and chemical profiling of Usnea cornuta Korb
Source: PLoS One. 2026 Jun 12;21(6):e0351423. doi: 10.1371/journal.pone.0351423 (PMC13262885; doi:10.1371/journal.pone.0351423)
Supplement: S1 File — S2 Fig. Standard curve of DPPH inhibition by Quercetin. S3 Fig. α-Glucosidase inhibition activity of Acarbose. S4 Fig. Ramachandran plot of protein (PDB ID: 3A4A). S5 Fig. Changes in the binding free energy of different protein adducts with (a) protein-menegazziaic acid and (b) acarbose, red indicates the moving average. S6 Fig. Residues contribution in binding energies (kcal/mol) of complexs. (a) menegazziaic acid and (b) acarbose complexes. S1 Table. Zone of inhibition of crude extract of U. cornuta against bacteria strain. S2 Table. Identification of metabolites in lichen Usnea cornuta by LC-MS. S3 Table. Mass spectra chromatogram of 11 compounds present in U. cornuta. S4 Table. Change in binding energies (kcal/mol) of complex with different components. (a) menegazziaic acid and (b) acarbose complexes. S5 Table. Drug-Likeness Properties of menegazziaic acid and acarbose through the Swiss ADME Portal. (ZIP) [file pone.0351423.s001.zip › Supporting Information/S5 Table.docx]

**S4 Table. Drug-Likeness Properties of menegazziaic acid and acarbose through the Swiss ADME Portal**

| **Compound Name** | **Num. rotatable bonds** | **Num. H-bond acceptors** | **Num. H-bond Donors** | **TPSA** | **GI absorption** | **BBB Permeant** | **Lipinski’s rule of five** |
| --- | --- | --- | --- | --- | --- | --- | --- |
| **Menegazziaic acid** | 1 | 9 | 3 | 131.75 | High | No | Yes; 0 violation |
| **Acarbose** | 9 | 19 | 14 | 321.17 | Low | No | No; 3 violations |
