# Supplementary material for: Antioxidant, antibacterial, in vitro, and in silico α-glucosidase inhibition activities and chemical profiling of Usnea cornuta Korb
Source: PLoS One. 2026 Jun 12;21(6):e0351423. doi: 10.1371/journal.pone.0351423 (PMC13262885; doi:10.1371/journal.pone.0351423)
Supplement: Attachment — Submitted filename: Review report PLOS ONE.docx [file pone.0351423.s002.docx]

1. The manuscript contains several grammatical and typographical errors that should be carefully revised.
2. The study appears to lack sufficient novelty; the authors are encouraged to clearly highlight the innovative aspects of their work.
3. In the introduction section, the rationale for selecting U. cornuta is not provided. The authors should explain why this plant was chosen—particularly if it has any ethnobotanical relevance for the treatment of diabetes.
4. In the Materials and Methods section, the description of the LC/MS methodology and instrumental details is insufficient and should be expanded for clarity and reproducibility.
5. Alpha-glucosidase is a catalytic enzyme with Asp518 and Asp616 serving as the catalytic dyad. None of the selected compounds showed interactions with this catalytic dyad. Therefore, it is unclear how the authors conclude that these compounds inhibit alpha-glucosidase, especially in the absence of supporting in vitro or in vivo assays.
6. Validation of the docking methodology has not been described and should be included to strengthen the reliability of the computational results.
7. The molecular dynamics simulations have only been qualitatively analyzed. To provide more meaningful insights, the authors should include quantitative analyses such as free energy landscape and PCA (principal component analysis) to assess conformational changes in the protein.
8. Although the dichloromethane (DCM) extract showed stronger alpha-glucosidase inhibition compared to the ethyl acetate extract, the authors selected the ethyl acetate extract for LC/MS analysis. The rationale for this choice should be clarified.
9. A comparison of the current findings with previously published literature is lacking and should be included to position the study within the existing body of research.
10. In the Results and Discussion section, only the outcomes of TPC, TFC, antioxidant, and alpha-glucosidase inhibition assays are presented. A deeper discussion of the significance and implications of these findings would strengthen the manuscript.
