# Supplementary material for: Antioxidant, antibacterial, in vitro, and in silico α-glucosidase inhibition activities and chemical profiling of Usnea cornuta Korb
Source: PLoS One. 2026 Jun 12;21(6):e0351423. doi: 10.1371/journal.pone.0351423 (PMC13262885; doi:10.1371/journal.pone.0351423)
Supplement: Attachment — Submitted filename: Response to Reviewers.docx [file pone.0351423.s003.docx]

To, 12/29/2025

The Academic Editor

**PLOS ONE**

Subject: Responses to the comments and suggestions

Dear Editor,

Herein, we are submitting our revised research manuscript entitled **"Antioxidant, antibacterial, *in vitro,* and *in silico* α-glucosidase inhibition activities and chemical profiling of *Usnea cornuta* Korb"** for publication in your esteemed journal, **PLOS ONE.** We have addressed the reviewer’s comments with track changes to the best of our knowledge and effort. We want to express our sincere gratitude for the insightful suggestions and for providing ample time for a thorough revision.

**Comments**

Introduction:

1. The introduction presents important content but is excessively long in some parts and superficial in others.

Example: there is meticulous detailing of chemical classes of lichens, but little effort in highlighting the specific scientific gap that the study intends to fill.

2. Reduce overly long taxonomic and chemical descriptions.
3. Emphasize the novelty of the study.

**Response:** Thank you for your comments. We have revised introduction part according to your suggestion.

Methodology:

4. The authors should include information on the collection and authentication of the biological material. The voucher number of the deposit in the herbarium should be included.
5. Full name of the taxonomist who performed the authentication.
6. Geographic coordinates of the collection (if applicable).

**Response**: Thank you for your comments. We have included the voucher code, the taxonomist’s name, and the geographic coordinates.

Extraction and fractionation process.

6.1 Describe the yield (%) of each fraction, not just the crude extract.
6.2 Report storage conditions and temperature of the fractions.

**Response:** We have specified each fraction in the results section and included the storage conditions and temperature in the Methodology section. Thank you.

The description of the antioxidant and α-glucosidase assays lacks operational information:

6.3 final reagent concentration
6.4 standard curves used
6.5 number of replicates
6.6 IC_50_ calculation method

**Response:** We have included all the operational details in the manuscript. Thank you.

Antibacterial assays

6.7 Report all strains used (ATCC, clinical isolate, etc.)
6.8 Report positive and negative controls used.
6.9 Describe in detail the criteria for sensitivity classification

**Response:** We have brought up all the suggestions that you have given us in manuscript. Thank You.

7. Molecular docking and molecular dynamics
   7.1 software and version
   7.2 field strength
   7.3 number of poses generated
   7.4 total simulation time (ns)
   7.5 equilibrium parameters
   7.6 description of cluster analysis

**Response:** We have added all the above detailed methodology section.

Results

8. IC_50_ values should always include unit, standard error, and number of replicates.
9. A table with all the results would be useful to facilitate comparison.
10. Discussion of compound menegazziaic acid
    10.1 The manuscript highlights this compound as a “hit”, but does not clearly demonstrate:
    10.2 Whether it was actually identified in the sample
    10.3 Whether it is present in large quantities

**Response:** We have incorporated all the suggestions you provided into the manuscript. Thank you.

The authors should explain/ justify whether docking was performed for all identified compounds or only for selected ones. They should also justify the selection of compounds.

11. LC-MS Data
    11.1 The authors should include the chromatograms of the identified compounds.

**Response:** We have justified the rationale for the selection of compounds in the manuscript and included the chromatogram figures of the identified compounds in the supplementary file. Thank you.

Discussions

12. There is an excess of quantitative comparison with other lichens species without a consolidated critical analysis.
13. The mechanistic explanations are superficial and, in some passages, speculative.
14. The role of specific compounds (usnic acid, salazinic acid, constrictive acid, etc.) should be better correlated with:
    14.1 the most active fractions
    14.2 docking results
    14.3 enzymatic and antibacterial activities
15. It is recommended to reorganize the discussion into thematic subsections:

Chemical Composition

Antioxidant activity

α-glucosidase activity

Antibacterial activity

Integration within silico data

Pharmacological perspectives

**Response:** All the suggestions have been addressed and incorporated into the manuscript. Thank you.

**Journal Requirements:**

1. Please ensure that your manuscript meets PLOS ONE’s style requirements, including those for file naming. We note that the grant information you provided in the ‘Funding Information’ and ‘Financial Disclosure’ sections do not match. Please state what role the funders took in the study. Please ensure that you refer to Figure 10 in your text as, if accepted, production will need this reference to link the reader to the figure. We note that Figure 1 in your submission contains copyrighted images. If the reviewer comments included a recommendation to cite specific previously published works, please review and evaluate these publications to determine whether they are relevant and should be cited.

**Response:** Thank you so much for your suggestions. We have mentioned the points that journal required in manuscript and remove the figure 1 from manuscript.

**Reviewer 1 comments:**

1. The manuscript contains several grammatical and typographical errors that should be carefully revised.

**Response:** We have carefully corrected several grammatical and typographical errors in the manuscript. Thank you.

The study appears to lack sufficient novelty; the authors are encouraged to clearly highlight the innovative aspects of their work.

**Response:** We have mentioned the novelty in the manuscript. Thank You.

2. In the introduction section, the rationale for selecting *U. cornuta* is not provided. The authors should explain why this plant was chosen, particularly if it has any ethnobotanical relevance for the treatment of diabetes.

**Response:** We have explained the rationale for selecting this lichen in the introduction section. Thank you.

4. In the Materials and Methods section, the description of the LC/MS methodology and instrumental details is insufficient and should be expanded for clarity and reproducibility.

**Response:** We have described the LC/MS methodology and instrumental details in detail and expanded the section to improve clarity and reproducibility. Thank you.

5. Alpha-glucosidase is a catalytic enzyme with Asp518 and Asp616 serving as the catalytic dyad. None of the selected compounds showed interactions with this catalytic dyad. Therefore, it is unclear how the authors conclude that these compounds inhibit alpha-glucosidase, especially in the absence of supporting in vitro or in vivo assays.

**Response:** We have added discussion section.

6. Validation of the docking methodology has not been described and should be included to strengthen the reliability of the computational results.

**Response:** We have added validation of the docking protocol in the methodology section.
7. The molecular dynamics simulations have only been qualitatively analyzed. To provide more meaningful insights, the authors should include quantitative analyses such as free energy landscape and PCA (principal component analysis) to assess conformational changes in the protein.

**Response:** We have added PCA, DCCM, and free energy landscape in the results section.

8. Although the dichloromethane (DCM) extract showed stronger alpha-glucosidase inhibition compared to the ethyl acetate extract, the authors selected the ethyl acetate extract for LC/MS analysis. The rationale for this choice should be clarified.

**Response:** Although the dichloromethane (DCM) fraction exhibited comparatively stronger α-glucosidase inhibitory activity (IC₅₀ = 2.59 ± 2.23 µg/mL) than the ethyl acetate fraction (IC₅₀ = 3.72 ± 0.64 µg/mL), the ethyl acetate extract was selected for LC–MS analysis for several reasons. The ethyl acetate is known to preferentially extract medium-polarity bioactive compounds, such as flavonoids and phenolics, which are commonly associated with antidiabetic and antioxidant activities and are more amenable to LC–MS characterization. Additionally, ethyl acetate fraction demonstrated more consistent inhibitory activity, as reflected by its lower standard error mean, indicating better reproducibility. In contrast, the DCM fraction showed greater variability, suggesting a heterogeneous composition or the presence of unstable constituents.

9. A comparison of the current findings with previously published literature is lacking and should be included in the position of the study within the existing body of research.

**Response:** We have cited previously published literature and clarified the position of our study within the existing body of research. Thank you.

10. In the Results and Discussion section, only the outcomes of TPC, TFC, antioxidant, and alpha-glucosidase inhibition assays are presented. A deeper discussion of the significance and implications of these findings would strengthen the manuscript.

**Response:** We have mentioned a deeper discussion of the significance and implications of these findings to strengthen the manuscript. Thank You.

Reviewer #2:

The manuscript presents an introductory study on Usnea cornuta. Below are a few suggestions for improvement:

1. Section 2.2: please add a voucher specimen.

**Response**: We have mentioned the voucher specimen. Thank You.

2. Why did the authors use quercetin as reference standard in antioxidant assay while there are some commonly used standards such as vitamin C and BHT?

**Response:** Quercetin was used as the reference standard because it is a natural flavonoid with phenolic structure and antioxidant mechanisms like the phenolic compounds present in *Usnea cornuta*. Compared to vitamin C, which is water soluble, and BHT, which is synthetic and lipid soluble, quercetin provides a more appropriate and reliable benchmark for evaluating natural antioxidant activity.

3. Please add statistical analysis.

**Response:** We have mentioned the additional statistical analysis. Thank You.

4. Table 3: correct quercetin into acarbose for the glucosidase results.

**Response:** We have corrected it according to your suggestion. Thank You.

5. Please detail information about the Shimadzu LC-MS 2020 system. Is it a single quad or high-resolution mass spectrometry system?

**Response:** The Shimadzu LCMS-2020 is a single quadrupole LC–MS system, not a high-resolution mass spectrometer. It uses a single quadrupole mass analyzer with unit mass resolution and is designed for routine qualitative screening and quantitative analysis. The system typically operates over a mass range of approximately m/z 10–2000 and supports ionization techniques such as ESI and APCI. Thank You.

6. Please add a representative LCMS chromatogram in the manuscript or supplementary data.

**Response:** We have added the LCMS chromatogram to supplementary data. Thank You.

7. Table S2: Are these all the metabolites in lichen Usnea cornuta tentatively identified by LC-MS?

**Response:** Yes, Table S2 contained all the metabolites in lichen Usnea cornuta tentatively identified by LC-MS and we have mentioned their individual chromatograph. Thank You.

8. Please add more discussion on antioxidant and α-glucosidase inhibition activity of the extracts and compare with results of other Usnea species.

**Response:** We have added more discussion on antioxidant and alpha-glucosidase inhibition activity of the extracts and compare with results of other Usnea species. Thank You.
