# Supplementary material for: Antioxidant, antibacterial, in vitro, and in silico α-glucosidase inhibition activities and chemical profiling of Usnea cornuta Korb
Source: PLoS One. 2026 Jun 12;21(6):e0351423. doi: 10.1371/journal.pone.0351423 (PMC13262885; doi:10.1371/journal.pone.0351423)
Supplement: Attachment — Submitted filename: Response_to_Reviewers_auresp_2.docx [file pone.0351423.s004.docx]

To, 04/28/2026

The Academic Editor

**PLOS ONE**

Subject: Responses to the comments and suggestions

Dear Editor,

Herein, we are submitting our revised research manuscript entitled **"Antioxidant, antibacterial, *in vitro,* and *in silico* α-glucosidase inhibition activities and chemical profiling of *Usnea cornuta* Korb"** for publication in your esteemed journal, **PLOS ONE.** We have addressed the reviewer’s comments with track changes to the best of our knowledge and effort. We want to express our sincere gratitude for the insightful suggestions and for taking the time to thoroughly revise.

**Reviewer 1 comments:**

1. The Sentence, “This method was used to assess alpha glucosidase inhibition activity found in *U.corunata* using p-NPG substrate by slight modification” in the methodology section of the invitro glucosidase assay is unclear and needs to be rewritten for clarity.

**Response:** Thank you for your valuable comment. The sentence has been revised for clarity.

2. The increment of the solvent composition in the gradient flow method has not been clearly illustrated.

**Response:** Thank you for your insightful comment. We agree that the gradient elution program was not clearly described in the original manuscript. The methods section has been revised to explicitly illustrate changes in solvent composition over time.

3. Identification of metabolites based solely on the retention time and mass spectral data isnot sufficiently accurate.

**Response:** We have mentioned in comment no. 4.

4. Although MestReNova software is widely used for NME Data analysis, it is not an appropriate tool for the analysis of high resolution mass spectrometry data. Moreover, there is a possibility of data loss during file conversion to other formats. Why did the authors not use the original Shimadzu software for LC/MS data analysis?

**Response:** Thank you for your remarkable comment. We sincerely apologize for the lack of clarity in the presentation of LC-MS data. At the time of analysis, the samples were sent abroad for LC-MS analysis, and only the raw data were provided to us. The study was conducted during the initial stage of our training, and MestReNova software available under a limited institutional license was used for data processing. Due to resource constraints at our institution, access to additional analytical software was not available. Although adduct ion peaks and fragments ion information were generated during data processing in MestReNova, these details were not included in the original manuscript. We acknowledge that inclusion of this information would strengthen the data interpretation. Unfortunately, we don’t have access to an active license of the software to reprocess additional outputs at this stage. We appreciate the reviewer understanding and have revised the manuscript where possible to improve clarity and completeness of the reported data.

5. The method used to validate LC/MS data has not been described.

**Response:** Thank you for this valuable comment. We agree that clarification regarding LC/MS data validation is important. In this study, compound identification was performed using a combination of chromatographic and mass spectrometric parameters, including retention time, m/z values, and mass spectral fragmentation patterns. The obtained data were further analyzed using MestReNova software for spectral interpretation.

In addition, tentative compound identification was supported by comparison of the observed mass spectral features with available literature data and reported compounds. No authentic reference standards were used; therefore, all identified constituents should be considered as putatively assigned based on LC/MS data interpretation.

6. Out of 11 identified compounds, only 6 were selected for molecular docking studies. Why were the remaining compounds excluded? Are these compounds completely new, or was there another reason for their omission?

**Response:** Thank you for your suggestion. We have selected 6 compounds for docking because their chemical structures are available in the PubChem database (not others).

7. The citations used in the methodology sections for molecular docking and molecular dynamics simulations are not original. Please cite the appropriate original references.

**Response:** We have carefully revised the manuscript and added proper original citations and references.

8. The presentation of the percentage yield results is unclear and needs correction.

**Response:** Thank you for this helpful comment. We agree that the presentation of the percentage yield data was unclear in the original manuscript. The table and corresponding text have been revised for clarity and proper formatting to improve readability.

9. The value of total flavonoids content (TFC) reported in the results section and table 2 are inconsistent; Please correct this discrepancy.

**Response:** Thank you for carefully identifying this inconsistency. We apologize for the error in the reported total flavonoid content (TFC) values. The results section and Table 2 have been thoroughly checked and corrected to ensure consistency between the text and tabulated data.

10. Parameters such as radius of gyration, solvent accessible surface area, and root mean square fluctuations are not sufficiently described, and no comparison with previously published studies has been provided.

**Response:** Thank you for your insightful suggestion. We have now added a comparison with previously published studies in the results section.

11. Although the authors have included DCCM plots, free energy landscapes, and PCA analyses, the discussion and interpretation of these results are completely lacking without proper interpretation; the inclusion of these results does not add meaningful value to the study.

**Response:** We appreciate your response. We have substantially revised the Results and Discussion part of the manuscript and added proper interpretation of PCA, DCCM, and FEL.
