# Supplementary material for: Antioxidant, antibacterial, in vitro, and in silico α-glucosidase inhibition activities and chemical profiling of Usnea cornuta Korb
Source: PLoS One. 2026 Jun 12;21(6):e0351423. doi: 10.1371/journal.pone.0351423 (PMC13262885; doi:10.1371/journal.pone.0351423)
Supplement: Peer review history

# Peer review: Decision Letter 0

**DOI:** 10.1371/journal.pone.0351423.r001

**Authors:** Jorddy Neves Cruz

14 Nov 2025

PONE-D-25-39054

Antioxidant, antibacterial, in vitro, and in silico α-glucosidase inhibition activities and chemical profiling of Usnea cornuta Korb

PLOS ONE

Dear Dr. Adhikari,

Thank you for submitting your manuscript to PLOS ONE. After careful consideration, we feel that it has merit but does not fully meet PLOS ONE’s publication criteria as it currently stands. Therefore, we invite you to submit a revised version of the manuscript that addresses the points raised during the review process.

=============================================================

The study combines in vitro assays, LC-MS, molecular docking, and molecular dynamics, configuring a multidimensional approach. The overall structure of the manuscript is clear, however, there are several issues that need to be reviewed.

# Introduction

1. The introduction presents important content, but is excessively long in some parts and superficial in others.

Example: there is meticulous detailing of chemical classes of lichens, but little effort in highlighting the specific scientific gap that the study intends to fill.

2. Reduce overly long taxonomic and chemical descriptions.

3. Emphasize the novelty of the study.

# Methodology

4. The authors should include information on the collection and authentication of the biological material. The voucher number of the deposit in the herbarium should be included.

5. Full name of the taxonomist who performed the authentication.

6. Geographic coordinates of the collection (if applicable).

7. Extraction and fractionation process.

7.1 Describe the yield (%) of each fraction, not just the crude extract.

7.2 Report storage conditions and temperature of the fractions.

8. The description of the antioxidant and α-glucosidase assays lacks operational information:

8.1 final reagent concentration

8.2 standard curves used

8.3 number of replicates

8.4 IC₅₀ calculation method

9. Antibacterial assays

9.1 Report all strains used (ATCC, clinical isolate, etc.)

9.2 Report positive and negative controls used.

9.3 Describe in detail the criteria for sensitivity classification

10. Molecular docking and molecular dynamics

10.1 software and version

10.2 field strength

10.3 number of poses generated

10.4 total simulation time (ns)

10.5 equilibrium parameters

10.6 description of cluster analysis

# Results

11. IC₅₀ values should always include unit, standard error, and number of replicates.

12. A table with all the results would be useful to facilitate comparison.

13. Discussion of the compound menegazziaic acid

13.1 The manuscript highlights this compound as a "hit," but does not clearly demonstrate:

13.2 whether it was actually identified in the sample

13.3 whether it is present in large quantities

14. The authors should explain/justify whether docking was performed for all identified compounds or only for selected ones. They should also justify the selection of compounds.

15. LC-MS Data

15.1 The authors should include the chromatograms of the identified compounds.

# Discussions

16. There is an excess of quantitative comparison with other lichen species without a consolidated critical analysis.

17. The mechanistic explanations are superficial and, in some passages, speculative.

18. The role of specific compounds (usnic acid, salazinic acid, constrictive acid, etc.) should be better correlated with:

18.1 the most active fractions

18.2 docking results

18.3 enzymatic and antibacterial activities

19. It is recommended to reorganize the discussion into thematic subsections:

Chemical composition

Antioxidant activity

α-glucosidase activity

Antibacterial activity

Integration with in silico data

Pharmacological perspectives

=============================================================

Please submit your revised manuscript by Dec 29 2025 11:59PM. If you will need more time than this to complete your revisions, please reply to this message or contact the journal office at plosone@plos.org. When you're ready to submit your revision, log on to https://www.editorialmanager.com/pone/ and select the 'Submissions Needing Revision' folder to locate your manuscript file.

Please include the following items when submitting your revised manuscript:

- A rebuttal letter that responds to each point raised by the academic editor and reviewer(s). You should upload this letter as a separate file labeled 'Response to Reviewers'.
- A marked-up copy of your manuscript that highlights changes made to the original version. You should upload this as a separate file labeled 'Revised Manuscript with Track Changes'.
- An unmarked version of your revised paper without tracked changes. You should upload this as a separate file labeled 'Manuscript'.

If you would like to make changes to your financial disclosure, please include your updated statement in your cover letter. Guidelines for resubmitting your figure files are available below the reviewer comments at the end of this letter.

If applicable, we recommend that you deposit your laboratory protocols in protocols.io to enhance the reproducibility of your results. Protocols.io assigns your protocol its own identifier (DOI) so that it can be cited independently in the future. For instructions see: https://journals.plos.org/plosone/s/submission-guidelines#loc-laboratory-protocols. Additionally, PLOS ONE offers an option for publishing peer-reviewed Lab Protocol articles, which describe protocols hosted on protocols.io. Read more information on sharing protocols at https://plos.org/protocols?utm_medium=editorial-email&utm_source=authorletters&utm_campaign=protocols.

We look forward to receiving your revised manuscript.

Kind regards,

Jorddy Neves Cruz

Academic Editor

PLOS ONE

Journal Requirements:

When submitting your revision, we need you to address these additional requirements.

1. Please ensure that your manuscript meets PLOS ONE's style requirements, including those for file naming. The PLOS ONE style templates can be found at https://journals.plos.org/plosone/s/file?id=wjVg/PLOSOne_formatting_sample_main_body.pdf and https://journals.plos.org/plosone/s/file?id=ba62/PLOSOne_formatting_sample_title_authors_affiliations.pdf

2. We note that the grant information you provided in the ‘Funding Information’ and ‘Financial Disclosure’ sections do not match.

When you resubmit, please ensure that you provide the correct grant numbers for the awards you received for your study in the ‘Funding Information’ section.

3. Thank you for stating the following financial disclosure:

The University Grant Commission provides financial support to carry out the research work with the reference number MRS-78-79-S&T-38.

Please state what role the funders took in the study. If the funders had no role, please state: "The funders had no role in study design, data collection and analysis, decision to publish, or preparation of the manuscript."

If this statement is not correct you must amend it as needed.

Please include this amended Role of Funder statement in your cover letter; we will change the online submission form on your behalf.

4. Please ensure that you refer to Figure 10 in your text as, if accepted, production will need this reference to link the reader to the figure.

5. We note that Figure 1 in your submission contain copyrighted images. All PLOS content is published under the Creative Commons Attribution License (CC BY 4.0), which means that the manuscript, images, and Supporting Information files will be freely available online, and any third party is permitted to access, download, copy, distribute, and use these materials in any way, even commercially, with proper attribution. For more information, see our copyright guidelines: http://journals.plos.org/plosone/s/licenses-and-copyright.

We require you to either (1) present written permission from the copyright holder to publish these figures specifically under the CC BY 4.0 license, or (2) remove the figures from your submission:

a. You may seek permission from the original copyright holder of Figure 1 to publish the content specifically under the CC BY 4.0 license.

We recommend that you contact the original copyright holder with the Content Permission Form (http://journals.plos.org/plosone/s/file?id=7c09/content-permission-form.pdf) and the following text:

“I request permission for the open-access journal PLOS ONE to publish XXX under the Creative Commons Attribution License (CCAL) CC BY 4.0 (http://creativecommons.org/licenses/by/4.0/). Please be aware that this license allows unrestricted use and distribution, even commercially, by third parties. Please reply and provide explicit written permission to publish XXX under a CC BY license and complete the attached form.”

Please upload the completed Content Permission Form or other proof of granted permissions as an "Other" file with your submission.

In the figure caption of the copyrighted figure, please include the following text: “Reprinted from [ref] under a CC BY license, with permission from [name of publisher], original copyright [original copyright year].”

b. If you are unable to obtain permission from the original copyright holder to publish these figures under the CC BY 4.0 license or if the copyright holder’s requirements are incompatible with the CC BY 4.0 license, please either i) remove the figure or ii) supply a replacement figure that complies with the CC BY 4.0 license. Please check copyright information on all replacement figures and update the figure caption with source information. If applicable, please specify in the figure caption text when a figure is similar but not identical to the original image and is therefore for illustrative purposes only.

6. If the reviewer comments include a recommendation to cite specific previously published works, please review and evaluate these publications to determine whether they are relevant and should be cited. There is no requirement to cite these works unless the editor has indicated otherwise.

[Note: HTML markup is below. Please do not edit.]

Reviewers' comments:

Reviewer's Responses to Questions

**Comments to the Author**

1. Is the manuscript technically sound, and do the data support the conclusions?

The manuscript must describe a technically sound piece of scientific research with data that supports the conclusions. Experiments must have been conducted rigorously, with appropriate controls, replication, and sample sizes. The conclusions must be drawn appropriately based on the data presented.

Reviewer #1: Yes

Reviewer #2: Partly

**********

2. Has the statistical analysis been performed appropriately and rigorously?

Reviewer #1: No

Reviewer #2: N/A

**********

3. Have the authors made all data underlying the findings in their manuscript fully available?

The PLOS Data policy requires authors to make all data underlying the findings described in their manuscript fully available without restriction, with rare exception (please refer to the Data Availability Statement in the manuscript PDF file). The data should be provided as part of the manuscript or its supporting information, or deposited to a public repository. For example, in addition to summary statistics, the data points behind means, medians and variance measures should be available. If there are restrictions on publicly sharing data—e.g. participant privacy or use of data from a third party—those must be specified.

Reviewer #1: Yes

Reviewer #2: No

**********

4. Is the manuscript presented in an intelligible fashion and written in standard English?

PLOS ONE does not copyedit accepted manuscripts, so the language in submitted articles must be clear, correct, and unambiguous. Any typographical or grammatical errors should be corrected at revision, so please note any specific errors here.

Reviewer #1: No

Reviewer #2: Yes

**********

5. Review Comments to the Author

Please use the space provided to explain your answers to the questions above. You may also include additional comments for the author, including concerns about dual publication, research ethics, or publication ethics. (Please upload your review as an attachment if it exceeds 20,000 characters)

Reviewer #1: 1. The manuscript contains several grammatical and typographical errors that should be carefully revised.

2. The study appears to lack sufficient novelty; the authors are encouraged to clearly highlight the innovative aspects of their work.

3. In the introduction section, the rationale for selecting U. cornuta is not provided. The authors should explain why this plant was chosen—particularly if it has any ethnobotanical relevance for the treatment of diabetes.

4. In the Materials and Methods section, the description of the LC/MS methodology and instrumental details is insufficient and should be expanded for clarity and reproducibility.

5. Alpha-glucosidase is a catalytic enzyme with Asp518 and Asp616 serving as the catalytic dyad. None of the selected compounds showed interactions with this catalytic dyad. Therefore, it is unclear how the authors conclude that these compounds inhibit alpha-glucosidase, especially in the absence of supporting in vitro or in vivo assays.

6. Validation of the docking methodology has not been described and should be included to strengthen the reliability of the computational results.

7. The molecular dynamics simulations have only been qualitatively analyzed. To provide more meaningful insights, the authors should include quantitative analyses such as free energy landscape and PCA (principal component analysis) to assess conformational changes in the protein.

8. Although the dichloromethane (DCM) extract showed stronger alpha-glucosidase inhibition compared to the ethyl acetate extract, the authors selected the ethyl acetate extract for LC/MS analysis. The rationale for this choice should be clarified.

9. A comparison of the current findings with previously published literature is lacking and should be included to position the study within the existing body of research.

10. In the Results and Discussion section, only the outcomes of TPC, TFC, antioxidant, and alpha-glucosidase inhibition assays are presented. A deeper discussion of the significance and implications of these findings would strengthen the manuscript.

Reviewer #2: The manuscript presents an introductory study on Usnea cornuta. Below are a few suggestions for improvement:

- Section 2.2: please add a voucher specimen.

- Why did the authors use quercetin as reference standard in antioxidant assay while there are some commonly used standards such as vitamin C and BHT?

- Please add statistical analysis.

- Table 3: correct quercetin into acarbose for the glucosidase results.

- Please detail information about the Shimadzu LC-MS 2020 system. Is it a single quad or high resolution mass spectrometry system?

- Please add a representative LCMS chromatogram in the manuscript or supplementary data.

- Table S2: Are these all the metabolites in lichen Usnea cornuta tentatively identified by LC-MS?

- Please add more discussion on antioxidant and α-glucosidase inhibition activity of the extracts and compare with results of other Usnea species.

**********

6. PLOS authors have the option to publish the peer review history of their article (what does this mean?). If published, this will include your full peer review and any attached files.

If you choose “no”, your identity will remain anonymous but your review may still be made public.

**Do you want your identity to be public for this peer review?**  For information about this choice, including consent withdrawal, please see our Privacy Policy.

Reviewer #1: No

Reviewer #2: No

**********

[NOTE: If reviewer comments were submitted as an attachment file, they will be attached to this email and accessible via the submission site. Please log into your account, locate the manuscript record, and check for the action link "View Attachments". If this link does not appear, there are no attachment files.]

To ensure your figures meet our technical requirements, please review our figure guidelines: https://journals.plos.org/plosone/s/figures

You may also use PLOS’s free figure tool, NAAS, to help you prepare publication quality figures: https://journals.plos.org/plosone/s/figures#loc-tools-for-figure-preparation.

NAAS will assess whether your figures meet our technical requirements by comparing each figure against our figure specifications.

Attachment Submitted filename: Review report PLOS ONE.docx

# Author comment: Author response to Decision Letter 1

**DOI:** 10.1371/journal.pone.0351423.r002

30 Dec 2025

1. The manuscript contains several grammatical and typographical errors that should be carefully revised.

Response: We have carefully corrected several grammatical and typographical errors in the manuscript. Thank you.

The study appears to lack sufficient novelty; the authors are encouraged to clearly highlight the innovative aspects of their work.

Response: We have mentioned the novelty in the manuscript. Thank You.

2. In the introduction section, the rationale for selecting U. cornuta is not provided. The authors should explain why this plant was chosen, particularly if it has any ethnobotanical relevance for the treatment of diabetes.

Response: We have explained the rationale for selecting this lichen in the Introduction section. Thank you.

4. In the Materials and Methods section, the description of the LC/MS methodology and instrumental details is insufficient and should be expanded for clarity and reproducibility.

Response: We have described the LC/MS methodology and instrumental details in detail and expanded the section to improve clarity and reproducibility. Thank you.

5. Alpha-glucosidase is a catalytic enzyme with Asp518 and Asp616 serving as the catalytic dyad. None of the selected compounds showed interactions with this catalytic dyad. Therefore, it is unclear how the authors conclude that these compounds inhibit alpha-glucosidase, especially in the absence of supporting in vitro or in vivo assays.

Response: We have added discussion section.

6. Validation of the docking methodology has not been described and should be included to strengthen the reliability of the computational results.

Response: We have added validation of the docking protocol in the methodology section.

7. The molecular dynamics simulations have only been qualitatively analyzed. To provide more meaningful insights, the authors should include quantitative analyses such as free energy landscape and PCA (principal component analysis) to assess conformational changes in the protein.

Response: We have added PCA, DCCM, and free energy landscape in the results section.

8. Although the dichloromethane (DCM) extract showed stronger alpha-glucosidase inhibition compared to the ethyl acetate extract, the authors selected the ethyl acetate extract for LC/MS analysis. The rationale for this choice should be clarified.

Response: Although the dichloromethane (DCM) fraction exhibited comparatively stronger α-glucosidase inhibitory activity (IC₅₀ = 2.59 ± 2.23 µg/mL) than the ethyl acetate fraction (IC₅₀ = 3.72 ± 0.64 µg/mL), the ethyl acetate extract was selected for LC–MS analysis for several reasons. The ethyl acetate is known to preferentially extract medium-polarity bioactive compounds, such as flavonoids and phenolics, which are commonly associated with antidiabetic and antioxidant activities and are more amenable to LC–MS characterization. Additionally, ethyl acetate fraction demonstrated more consistent inhibitory activity, as reflected by its lower standard deviation, indicating better reproducibility. In contrast, the DCM fraction showed greater variability, suggesting a heterogeneous composition or the presence of unstable constituents.

9. A comparison of the current findings with previously published literature is lacking and should be included in the position of the study within the existing body of research.

Response: We have cited previously published literature and clarified the position of our study within the existing body of research. Thank you.

10. In the Results and Discussion section, only the outcomes of TPC, TFC, antioxidant, and alpha-glucosidase inhibition assays are presented. A deeper discussion of the significance and implications of these findings would strengthen the manuscript.

Response: We have mentioned a deeper discussion of the significance and implications of these findings to strengthen the manuscript. Thank You.

Attachment Submitted filename: Response to Reviewers.docx

# Peer review: Decision Letter 1

**DOI:** 10.1371/journal.pone.0351423.r003

**Authors:** Jorddy Neves Cruz

10 Mar 2026

PONE-D-25-39054R1

Antioxidant, antibacterial, in vitro, and in silico α-glucosidase inhibition activities and chemical profiling of Usnea cornuta Korb

PLOS One

Dear Dr. Adhikari,

Thank you for submitting your manuscript to PLOS ONE. After careful consideration, we feel that it has merit but does not fully meet PLOS ONE’s publication criteria as it currently stands. Therefore, we invite you to submit a revised version of the manuscript that addresses the points raised during the review process.

Please submit your revised manuscript by Apr 24 2026 11:59PM. If you will need more time than this to complete your revisions, please reply to this message or contact the journal office at plosone@plos.org. When you're ready to submit your revision, log on to https://www.editorialmanager.com/pone/ and select the 'Submissions Needing Revision' folder to locate your manuscript file.

Please include the following items when submitting your revised manuscript:

- A letter that responds to each point raised by the academic editor and reviewer(s). You should upload this letter as a separate file labeled 'Response to Reviewers'.
- A marked-up copy of your manuscript that highlights changes made to the original version. You should upload this as a separate file labeled 'Revised Manuscript with Track Changes'.
- An unmarked version of your revised paper without tracked changes. You should upload this as a separate file labeled 'Manuscript'.

If you would like to make changes to your financial disclosure, please include your updated statement in your cover letter. Guidelines for resubmitting your figure files are available below the reviewer comments at the end of this letter.

If applicable, we recommend that you deposit your laboratory protocols in protocols.io to enhance the reproducibility of your results. Protocols.io assigns your protocol its own identifier (DOI) so that it can be cited independently in the future. For instructions see: https://journals.plos.org/plosone/s/submission-guidelines#loc-laboratory-protocols. Additionally, PLOS ONE offers an option for publishing peer-reviewed Lab Protocol articles, which describe protocols hosted on protocols.io. Read more information on sharing protocols at https://plos.org/protocols?utm_medium=editorial-email&utm_source=authorletters&utm_campaign=protocols.

We look forward to receiving your revised manuscript.

Kind regards,

Jorddy Neves Cruz

Academic Editor

PLOS One

Journal Requirements:

If the reviewer comments include a recommendation to cite specific previously published works, please review and evaluate these publications to determine whether they are relevant and should be cited. There is no requirement to cite these works unless the editor has indicated otherwise.

[Note: HTML markup is below. Please do not edit.]

Reviewers' comments:

Reviewer's Responses to Questions

**Comments to the Author**

1. If the authors have adequately addressed your comments raised in a previous round of review and you feel that this manuscript is now acceptable for publication, you may indicate that here to bypass the “Comments to the Author” section, enter your conflict of interest statement in the “Confidential to Editor” section, and submit your "Accept" recommendation.

Reviewer #1: (No Response)

**********

2. Is the manuscript technically sound, and do the data support the conclusions?

The manuscript must describe a technically sound piece of scientific research with data that supports the conclusions. Experiments must have been conducted rigorously, with appropriate controls, replication, and sample sizes. The conclusions must be drawn appropriately based on the data presented.

Reviewer #1: Partly

**********

3. Has the statistical analysis been performed appropriately and rigorously?

Reviewer #1: No

**********

4. Have the authors made all data underlying the findings in their manuscript fully available?

The PLOS Data policy requires authors to make all data underlying the findings described in their manuscript fully available without restriction, with rare exception (please refer to the Data Availability Statement in the manuscript PDF file). The data should be provided as part of the manuscript or its supporting information, or deposited to a public repository. For example, in addition to summary statistics, the data points behind means, medians and variance measures should be available. If there are restrictions on publicly sharing data—e.g. participant privacy or use of data from a third party—those must be specified.

Reviewer #1: Yes

**********

5. Is the manuscript presented in an intelligible fashion and written in standard English?

PLOS ONE does not copyedit accepted manuscripts, so the language in submitted articles must be clear, correct, and unambiguous. Any typographical or grammatical errors should be corrected at revision, so please note any specific errors here.

Reviewer #1: No

**********

6. Review Comments to the Author

Please use the space provided to explain your answers to the questions above. You may also include additional comments for the author, including concerns about dual publication, research ethics, or publication ethics. (Please upload your review as an attachment if it exceeds 20,000 characters)

Reviewer #1: 1. The sentence, “This method was used to assess the α-glucosidase inhibition activity found in U. cornuta using the p-NPG substrate by slight modification,” in the methodology section of the in vitro glucosidase assay is unclear and needs to be rewritten for clarity.

2. The increment of the solvent composition in the gradient flow method has not been clearly illustrated.

3. Identification of metabolites based solely on retention time and mass spectral data is not sufficiently accurate.

4. Although MestReNova software is widely used for NMR data analysis, it is not an appropriate tool for the analysis of high-resolution mass spectrometry data. Moreover, there is a possibility of data loss during file conversion to other formats. Why did the authors not use the original Shimadzu software for LC/MS data analysis?

5. The method used to validate the LC/MS data has not been described.

6. Out of the 11 identified compounds, only 6 were selected for molecular docking studies. Why were the remaining compounds excluded? Are these compounds completely new, or was there another reason for their omission?

7. The citations used in the methodology sections for molecular docking and molecular dynamics simulations are not original. Please cite the appropriate original references.

8. The presentation of the percentage yield results is unclear and needs correction.

9. The values of total flavonoid content (TFC) reported in the Results section and Table 2 are inconsistent. Please correct this discrepancy.

10. Parameters such as radius of gyration, solvent-accessible surface area, and root mean square fluctuations are not sufficiently described, and no comparison with previously published studies has been provided.

11. Although the authors have included DCCM plots, free energy landscapes, and PCA analyses, the discussion and interpretation of these results are completely lacking. Without proper interpretation, the inclusion of these results does not add meaningful value to the study.

**********

7. PLOS authors have the option to publish the peer review history of their article (what does this mean?). If published, this will include your full peer review and any attached files.

If you choose “no”, your identity will remain anonymous but your review may still be made public.

**Do you want your identity to be public for this peer review?**  For information about this choice, including consent withdrawal, please see our Privacy Policy.

Reviewer #1: No

**********

[NOTE: If reviewer comments were submitted as an attachment file, they will be attached to this email and accessible via the submission site. Please log into your account, locate the manuscript record, and check for the action link "View Attachments". If this link does not appear, there are no attachment files.]

To ensure your figures meet our technical requirements, please review our figure guidelines: https://journals.plos.org/plosone/s/figures

You may also use PLOS’s free figure tool, NAAS, to help you prepare publication quality figures: https://journals.plos.org/plosone/s/figures#loc-tools-for-figure-preparation.

NAAS will assess whether your figures meet our technical requirements by comparing each figure against our figure specifications.

# Author comment: Author response to Decision Letter 2

**DOI:** 10.1371/journal.pone.0351423.r004

28 Apr 2026

1. The Sentence, “This method was used to assess alpha glucosidase inhibition activity found in U.corunata using p-NPG substrate by slight modification” in the methodology section of the invitro glucosidase assay is unclear and needs to be rewritten for clarity.

Response: Thank you for your valuable comment. The sentence has been revised for clarity.

2. The increment of the solvent composition in the gradient flow method has not been clearly illustrated.

Response: Thank you for your insightful comment. We agree that the gradient elution program was not clearly described in the original manuscript. The methods section has been revised to explicitly illustrate changes in solvent composition over time.

3. Identification of metabolites based solely on the retention time and mass spectral data isnot sufficiently accurate.

Response: We have mentioned in comment no. 4.

4. Although MestReNova software is widely used for NME Data analysis, it is not an appropriate tool for the analysis of high resolution mass spectrometry data. Moreover, there is a possibility of data loss during file conversion to other formats. Why did the authors not use the original Shimadzu software for LC/MS data analysis?

Response: Thank you for your remarkable comment. We sincerely apologize for the lack of clarity in the presentation of LC-MS data. At the time of analysis, the samples were sent abroad for LC-MS analysis, and only the raw data were provided to us. The study was conducted during the initial stage of our training, and MestReNova software available under a limited institutional license was used for data processing. Due to resource constraints at our institution, access to additional analytical software was not available. Although adduct ion peaks and fragments ion information were generated during data processing in MestReNova, these details were not included in the original manuscript. We acknowledge that inclusion of this information would strengthen the data interpretation. Unfortunately, we don’t have access to an active license of the software to reprocess additional outputs at this stage. We appreciate the reviewer understanding and have revised the manuscript where possible to improve clarity and completeness of the reported data.

5. The method used to validate LC/MS data has not been described.

Response: Thank you for this valuable comment. We agree that clarification regarding LC/MS data validation is important. In this study, compound identification was performed using a combination of chromatographic and mass spectrometric parameters, including retention time, m/z values, and mass spectral fragmentation patterns. The obtained data were further analyzed using MestReNova software for spectral interpretation.

In addition, tentative compound identification was supported by comparison of the observed mass spectral features with available literature data and reported compounds. No authentic reference standards were used; therefore, all identified constituents should be considered as putatively assigned based on LC/MS data interpretation.

6. Out of 11 identified compounds, only 6 were selected for molecular docking studies. Why were the remaining compounds excluded? Are these compounds completely new, or was there another reason for their omission?

Response: Thank you for your suggestion. We have selected 6 compounds for docking because their chemical structures are available in the PubChem database (not others).

7. The citations used in the methodology sections for molecular docking and molecular dynamics simulations are not original. Please cite the appropriate original references.

Response: We have carefully revised the manuscript and added proper original citations and references.

8. The presentation of the percentage yield results is unclear and needs correction.

Response: Thank you for this helpful comment. We agree that the presentation of the percentage yield data was unclear in the original manuscript. The table and corresponding text have been revised for clarity and proper formatting to improve readability.

9. The value of total flavonoids content (TFC) reported in the results section and table 2 are inconsistent; Please correct this discrepancy.

Response: Thank you for carefully identifying this inconsistency. We apologize for the error in the reported total flavonoid content (TFC) values. The results section and Table 2 have been thoroughly checked and corrected to ensure consistency between the text and tabulated data.

10. Parameters such as radius of gyration, solvent accessible surface area, and root mean square fluctuations are not sufficiently described, and no comparison with previously published studies has been provided.

Response: Thank you for your insightful suggestion. We have now added a comparison with previously published studies in the results section.

11. Although the authors have included DCCM plots, free energy landscapes, and PCA analyses, the discussion and interpretation of these results are completely lacking without proper interpretation; the inclusion of these results does not add meaningful value to the study.

Response: We appreciate your response. We have substantially revised the Results and Discussion part of the manuscript and added proper interpretation of PCA, DCCM, and FEL.

Attachment Submitted filename: Response_to_Reviewers_auresp_2.docx

# Peer review: Decision Letter 2

**DOI:** 10.1371/journal.pone.0351423.r005

**Authors:** Jorddy Neves Cruz

27 May 2026

Antioxidant, antibacterial, in vitro, and in silico α-glucosidase inhibition activities and chemical profiling of Usnea cornuta Korb

PONE-D-25-39054R2

Dear Dr. Adhikari,

We’re pleased to inform you that your manuscript has been judged scientifically suitable for publication and will be formally accepted for publication once it meets all outstanding technical requirements.

Within one week, you’ll receive an e-mail detailing the required amendments. When these have been addressed, you’ll receive a formal acceptance letter and your manuscript will be scheduled for publication.

An invoice will be generated when your article is formally accepted. Please note, if your institution has a publishing partnership with PLOS and your article meets the relevant criteria, all or part of your publication costs will be covered. Please make sure your user information is up-to-date by logging into Editorial Manager at Editorial Manager® and clicking the ‘Update My Information' link at the top of the page. For questions related to billing, please contact billing support.

If your institution or institutions have a press office, please notify them about your upcoming paper to help maximize its impact. If they’ll be preparing press materials, please inform our press team as soon as possible -- no later than 48 hours after receiving the formal acceptance. Your manuscript will remain under strict press embargo until 2 pm Eastern Time on the date of publication. For more information, please contact onepress@plos.org.

Kind regards,

Jorddy Neves Cruz

Academic Editor

PLOS One

Additional Editor Comments (optional):

Reviewers' comments:

Reviewer's Responses to Questions

**Comments to the Author**

1. If the authors have adequately addressed your comments raised in a previous round of review and you feel that this manuscript is now acceptable for publication, you may indicate that here to bypass the “Comments to the Author” section, enter your conflict of interest statement in the “Confidential to Editor” section, and submit your "Accept" recommendation.

Reviewer #1: All comments have been addressed

**********

2. Is the manuscript technically sound, and do the data support the conclusions?

The manuscript must describe a technically sound piece of scientific research with data that supports the conclusions. Experiments must have been conducted rigorously, with appropriate controls, replication, and sample sizes. The conclusions must be drawn appropriately based on the data presented.

Reviewer #1: Yes

**********

3. Has the statistical analysis been performed appropriately and rigorously?

Reviewer #1: Yes

**********

4. Have the authors made all data underlying the findings in their manuscript fully available?

The PLOS Data policy requires authors to make all data underlying the findings described in their manuscript fully available without restriction, with rare exception (please refer to the Data Availability Statement in the manuscript PDF file). The data should be provided as part of the manuscript or its supporting information, or deposited to a public repository. For example, in addition to summary statistics, the data points behind means, medians and variance measures should be available. If there are restrictions on publicly sharing data—e.g. participant privacy or use of data from a third party—those must be specified.

Reviewer #1: Yes

**********

5. Is the manuscript presented in an intelligible fashion and written in standard English?

PLOS ONE does not copyedit accepted manuscripts, so the language in submitted articles must be clear, correct, and unambiguous. Any typographical or grammatical errors should be corrected at revision, so please note any specific errors here.

Reviewer #1: Yes

**********

6. Review Comments to the Author

Please use the space provided to explain your answers to the questions above. You may also include additional comments for the author, including concerns about dual publication, research ethics, or publication ethics. (Please upload your review as an attachment if it exceeds 20,000 characters)

Reviewer #1: The manuscript is written in a well manner and maximum comments have been addressed. I have some minor suggestion and queries.

1. There are still some citation problems, Please use the original references.

2.The authors mentioned that only six metabolites were identified in the PubChem database. Does this imply that the remaining compounds are novel? Furthermore, how did the authors confirm the structures of the other compounds solely by comparing the MS peaks with metabolites reported from other lichen genera?

3. There is still only a qualitative interpretation of the DCCM and PCA analyses. The conformational changes occurring in the protein upon complex formation with the hit molecules have not been adequately explained.

**********

7. PLOS authors have the option to publish the peer review history of their article (what does this mean?). If published, this will include your full peer review and any attached files.

If you choose “no”, your identity will remain anonymous but your review may still be made public.

**Do you want your identity to be public for this peer review?**  For information about this choice, including consent withdrawal, please see our Privacy Policy.

Reviewer #1: No

**********

# Editor report: Acceptance letter

**DOI:** 10.1371/journal.pone.0351423.r006

**Authors:** Jorddy Neves Cruz

PONE-D-25-39054R2

PLOS One

Dear Dr. Adhikari,

I'm pleased to inform you that your manuscript has been deemed suitable for publication in PLOS One. Congratulations! Your manuscript is now being handed over to our production team.

At this stage, our production department will prepare your paper for publication. This includes ensuring the following:

* All references, tables, and figures are properly cited

* All relevant supporting information is included in the manuscript submission,

* There are no issues that prevent the paper from being properly typeset

You will receive further instructions from the production team, including instructions on how to review your proof when it is ready. Please keep in mind that we are working through a large volume of accepted articles, so please give us a few days to review your paper and let you know the next and final steps.

Lastly, if your institution or institutions have a press office, please let them know about your upcoming paper now to help maximize its impact. If they'll be preparing press materials, please inform our press team within the next 48 hours. Your manuscript will remain under strict press embargo until 2 pm Eastern Time on the date of publication. For more information, please contact onepress@plos.org.

You will receive an invoice from PLOS for your publication fee after your manuscript has reached the completed accept phase. If you receive an email requesting payment before acceptance or for any other service, this may be a phishing scheme. Learn how to identify phishing emails and protect your accounts at https://explore.plos.org/phishing.

If we can help with anything else, please email us at customercare@plos.org.

Thank you for submitting your work to PLOS ONE and supporting open access.

Kind regards,

PLOS ONE Editorial Office Staff

on behalf of

Dr. Jorddy Neves Cruz

Academic Editor

PLOS One
